# ATP-Independent Cooperative Binding of Yeast Isw1a to Bare and Nucleosomal DNA

**DOI:** 10.1371/journal.pone.0031845

**Published:** 2012-02-16

**Authors:** Anne De Cian, Elise Praly, Fangyuan Ding, Vijender Singh, Christophe Lavelle, Eric Le Cam, Vincent Croquette, Olivier Piétrement, David Bensimon

**Affiliations:** 1 Maintenance des Génomes, Microscopies Moléculaires et Bionanosciences, UMR 8126 CNRS and Université Paris Sud, Villejuif, France; 2 Laboratoire de Physique Statistique, Ecole Normale Supérieure, UMR 8550 CNRS, Paris, France; 3 The Wellcome Trust Biocentre, University of Dundee, Dundee, Scotland, United Kingdom; 4 Genome Dynamics and Regulation, CNRS UMR 7196, INSERM U565, Paris, France; National Cancer Institute, United States of America

## Abstract

Among chromatin remodeling factors, the ISWI family displays a nucleosome-enhanced ATPase activity coupled to DNA translocation. While these enzymes are known to bind to DNA, their activity has not been fully characterized. Here we use TEM imaging and single molecule manipulation to investigate the interaction between DNA and yeast Isw1a. We show that Isw1a displays a highly cooperative ATP-independent binding to and bridging between DNA segments. Under appropriate tension, rare single nucleation events can sometimes be observed and loop DNA with a regular step. These nucleation events are often followed by binding of successive complexes bridging between nearby DNA segments in a zipper-like fashion, as confirmed by TEM observations. On nucleosomal substrates, we show that the specific ATP-dependent remodeling activity occurs in the context of cooperative Isw1a complexes bridging extranucleosomal DNA. Our results are interpreted in the context of the recently published partial structure of Isw1a and support its acting as a “protein ruler” (with possibly more than one tick).

## Introduction

The eukaryotic genome is organized into chromatin, a dense nucleoproteic structure that provides a means of packaging DNA within the nucleus as well as a strategy to regulate the accessibility of DNA to proteins that need to contact it directly, such as transcription factors. The highly condensed structure of chromatin needs to be regulated throughout the cell cycle. For that purpose, eukaryotes have devised a number of strategies by which they can manipulate chromatin structure [1], [2]. These include the covalent alteration of chromatin through post-translational modifications of histones [3], the manipulation of the protein content of chromatin through the association of histone variants [4] and non-histone proteins [5] and the non-covalent alteration of chromatin structure by ATP-dependent chromatin-remodeling activities [6]. This approach is implemented by chromatin-remodeling factors, which use the energy of ATP hydrolysis to move histone octamers relative to DNA in order to make it accessible. These enzymes consist of a catalytic subunit with a region of homology to the yeast Snf2 protein that can be associated to a variable number of accessory subunits. These chromatin remodeling factors split into 24 distinct families depending on the sequence homology of their catalytic subunit [7]. The most studied factors belong to the Iswi, Swi2/Snf2, Mi-2, Chd, Ino80, Swr1 and Rad54 subfamilies [6], [8]. These complexes have been found to function in various processes ranging from the regulation of transcription and DNA replication or repair [9] to the maintenance of chromatin structure [10]; ISWI family members share this range of activity [11], [12], [13]. Remodelers also usually display different nucleosome positioning activities *in vitro* depending on the family, the catalytic subunit or the non-catalytic subunits associated [14], [15], [16].

Efforts have been made to get insight into the remodeling mechanisms by which these factors could exert their various functions and nucleosome repositioning activities [17]. Biochemical and structural data have led to a model where the remodelers first binds to extranucleosomal DNA and histone octamer and then translocate linker DNA into the nucleosome thanks to their ATPase activity. The translocation results in the formation of loops and/or torsional constraint, which by propagating over the histone octamer lead to a repositioning of the nucleosome [6], [18]. Single molecule techniques have recently allowed to re-address this model, supporting but also challenging some of its aspects [19]. Members of the Swi2/Snf2 family (namely RSC and SWI/SNF) were studied using magnetic [20] or optical [21], [22] tweezers. These single-molecule studies have shown that this remodelling factor act as single complex which translocates DNA in an inchworm-like model involving both twist and loop propagation (∼1 bp of twist for 10 bps of translocation).

In order to directly investigate the action of an ISWI family member on DNA, we have studied the behaviour of the yeast complex Isw1a on single DNA molecules using an approach similar to the one we formerly used to study the RSC complex [20] and other DNA-interacting proteins [23], [24], [25]. In absence of ATP and in contrast with RSC, which binds to DNA as an isolated complex, Isw1a exhibits strong binding cooperativity on bare DNA. Binding is initiated at positions where DNA can be bridged, e.g. apex of DNA loops or plectonemes. It then proceeds zipper-like by the cooperative binding of successive complexes on DNA, resulting in observed discrete changes in the DNA's extension (multiples of about 28 nm) and attributed to the bridging between nearby DNA segments. The large fluctuations in the molecule's extension observed in the compacted DNA suggest that the multi-proteins complex formed by Isw1a on DNA is very dynamic. The assembly of Isw1a on positively and negatively supercoiled DNA yields similar degrees of compaction, implying an absence of compensatory supercoils and suggesting that the DNA interacting with Isw1a is only weakly twisted, if at all. TEM imaging of Isw1a bound to linear and negatively supercoiled DNA confirm the cooperative assembly of the complex and its bridging between DNA segments. These complexes result from DNA-protein and protein-protein interactions and present the ability to form inter- or intra-molecular DNA bridging with a surprising ability to fold DNA molecules in two parts. This property results, on nucleosomal substrates, in the zipping of the external DNA arms. The ATP-dependent remodeling activity of Isw1a is observed in presence of these cooperative complexes and may be facilitated by them.

## Materials and Methods

### Protein purification

Isw1a complex was purified from a Ioc3-TAP (YTT1168) tagged yeast strains kindly provided by Toshio Tsukiyama. Yeast cells were grown at 30°C in 3× YPAD to an A600 of 2–2.5 and frozen by dropwise addition into liquid nitrogen. Yeast cells were harvested and lysed in a planetary grinder. The lysate was then thawed and purified by standard TAP protocols [26] using high stringency wash buffers (20 mM Na-Hepes (pH 7.5), 350 mM NaCl, 10% (v/v) glycerol, 0.1% (v/v) Tween-20, 1 mM 4-(2-Aminoethyl) benzenesulfonyl fluoride hydrochloride, 2.6 mM aprotinin, 2 µg/ml leupeptin, 1 µM pepstatin). The purified eluate was concentrated using a Centricon YM-50 concentrator (Millipore) to 200–250 µl and dialysed against wash buffer without protease inhibitors (and without Tween 20 for TEM experiments). Fractions were loaded and electrophoresed on a NuPAGE 4–12% Bis-Tris gel (Invitrogen) as per manufacturers instructions. The gel was stained with Instant Blue (Expedeon protein Solutions) for visualisation of protein bands (Figure S1).

### Sample preparation and magnetic trap experimental setup

Linear DNA molecules (∼3.6 kbp) modified at their extremities with digoxygenins at one end and biotins at the other, were synthesized as described previously [20]. These molecules were then attached at one end to the glass surface of a microscope flow chamber (previously coated with anti-digoxygenin) and at the other end to a 1 µm paramagnetic bead (DYNAL MyOne beads coated with streptavidin). Small magnets [27] were used to twist and pull on single DNA molecules attached to the beads. The DNA's extension was monitored by video microscopy of the tethered bead [28]. By tracking the bead's 3D position [27], [28], the mean extension ℓ_F_ = <z> of the molecule can be measured, with an error of ∼10 nm upon 1 s averaging. The horizontal motion of the bead *<δx^2^>* allows for the determination of the stretching force via the equipartition theorem *F = k_B_T* ℓ_F_ */<δx^2^>*. F was measured with 10% accuracy. To eliminate microscope drift, differential tracking was performed with a second bead glued to the surface. In the absence of ATP, the experiments with magnetic traps were performed in the following buffer: 10 mM TrisOAc pH∼7.9, 50 mM KCl, 0.1 mM DTT, 0.2% BSA. In the presence of ATP, 3 mM MgOAc was added to the preceding buffer. All experiments were performed at 29°C.

### Magnetic tweezers data processing

The acquisition of the molecule's extension ℓ_F_ is done with a CCD camera at 60 Hz. The raw data are then averaged over 0.5 s. To determine the change in extension consecutive to the binding or unbinding of a single Isw1a complex, as well as the values of τ_on_ and τ_off_, we fitted each telegraphic-like signal to a polygon defined by its six vertices: ℓ_i_,t_i_ (i = 1,…,6), as described elsewhere [20]. Here, we impose that three of the polygon's segments are horizontal (segments 1, 3 and 5) whereas two are vertical (segments 2, 4). Moreover, we impose that ℓ_1_ = ℓ_2_ = ℓ_5_ = ℓ_6_. The coordinates of the polygon vertices were adjusted to minimize the error (Chi-squared test). The increase/decrease in extension is then determined: δℓ_F_ = ℓ_3_−ℓ_2_ = ℓ_4_−ℓ_5_, the on time (resp. off time) are given by *t*_4_−*t*_3_ when ℓ_3_<ℓ_2_ (resp. ℓ_3_>ℓ_2_). To convert the measured variation in length δℓ_F_ into a cristallographic distance δℓ, we have to correct this value by the molecule's relative extension λ_WLC_ obtained from the Worm Like Chain (WLC) model [29]: δℓ = δℓ_F_/λ_WLC_ (at F = 1 pN, λ_WLC_≅0.85).

### DNA and chromatin preparation for TEM studies

pBR322 negatively supercoiled plasmid was purchased from Fermentas. Linear 3983 bps DNA was produced by enzymatic digestion of pBR322 by EcoRV and SspI (New England Biolabs) followed by further purification on gel filtration chromatography (Superose 6 column, GE-Healthcare) on FPCL SMART purifier (GE-Healthcare). For mononucleosome reconstitution, a 845 bps fragment containing the positioning sequence 601 was produced by PCR on pGEM3Z-601 template kindly provided by J. Widom using primers 5′-biotin-GAGTGCACCATATGCGGTGTGA-3′ and 5′-cy5-CCCAATACGCAAACCGCCTCTC-3′ followed by anion exchange chromatography (MiniQ column, GE-Healthcare). Chromatin was assembled on 845 bps DNA by exchange in high salt conditions with purified core particles (CP) from calf thymus and purified from free 146 bps fragments as described previously [30].

### TEM studies (binding and remodeling experiments) and data processing

Binding studies were carried out by mixing 750 nM (bp concentration) of DNA or nucleosomal substrates (which is equivalent to 0.89 nM of mononucleosomes on a 845 pbs substrate containing a 601 positioning sequence) with 0.5 µL of Isw1a (20-fold concentrated solutions or control buffer containing 20 mM Hepes-NaOH, pH 7.6, 350 mM NaCl and 15% glycerol) in 10 mM Tris-HCl pH 8, 30 mM KCl in a final volume of 10 µL. After 20 min incubation at 30°C, 5 µL of this reaction was deposited on a 600-mesh copper grid covered with a thin carbon film, activated by glow-discharge in the presence of pentylamine [31]. Grids were washed with aqueous 2% (w/v) uranyl acetate, dried and observed in the annular dark-field mode, using a Zeiss 902 transmission electron microscope. Images were captured at magnifications of 50,000×, 85000× and 140000× with a MegaviewIII CCD camera and iTEM software for acquisition (Olympus Soft Imaging Solution).

Remodeling activity was carried out as in the binding study except that 2 mM of Mg(OAc)_2_ and 75 µM of ATP (when present) were added to the reaction. When studying the effect of Isw1a bridging on remodeling, a preincubation of mononucleosome with Isw1a (10 min at 30°C) before addition of ATP and reaction for 20 min was done. Length analysis was done solely on mononucleosomal substrates using a graphic tablet and ImageJ software. Graphic representation and data analysis were performed using Excel and Prism 5.0 software.

## Results

**Figure 1:**
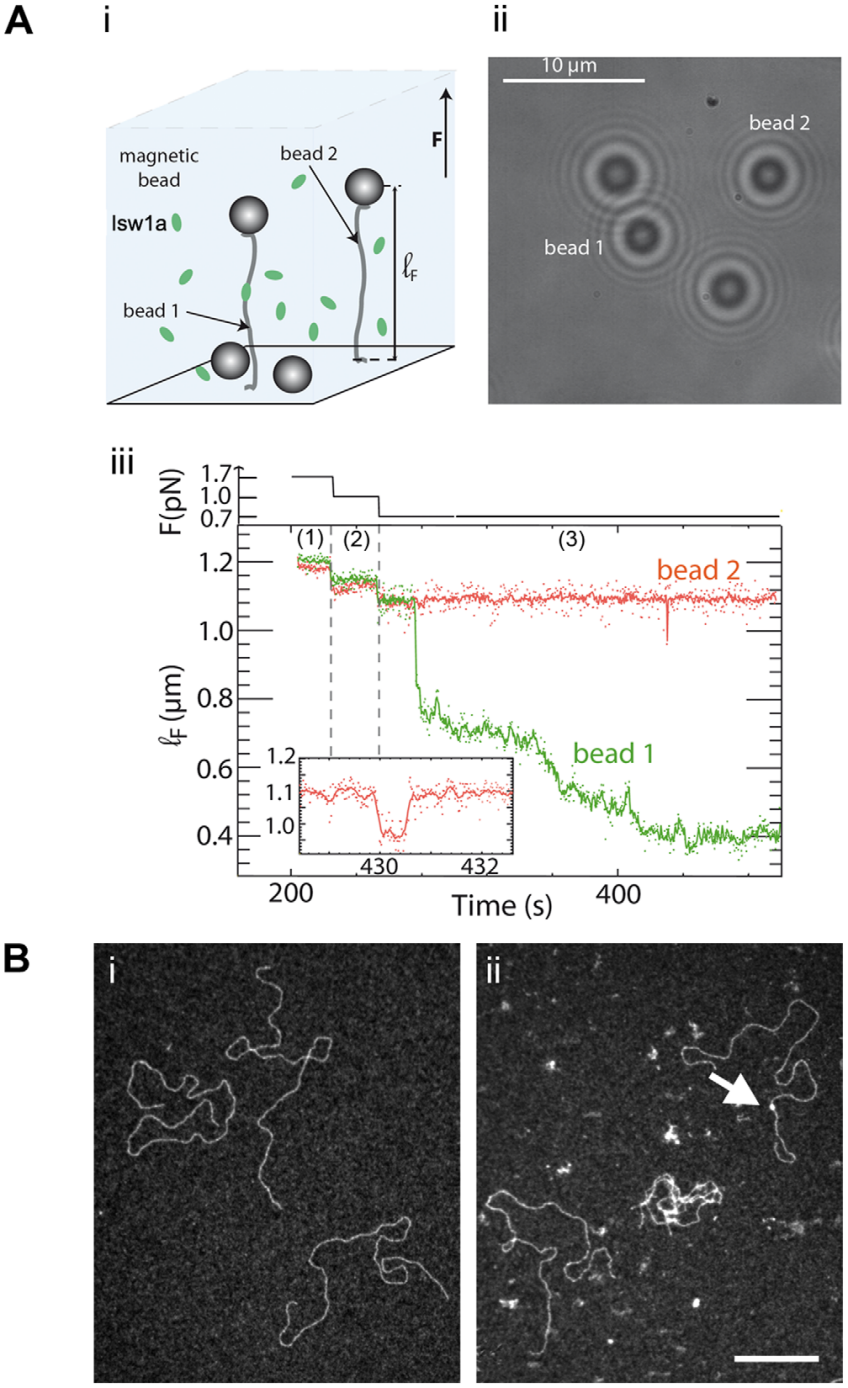
Cooperative binding of Isw1a to DNA. (A) (i) Illustration of the magnetic tweezers experiment: the extension (ℓ_F_∼1.2 µm) of two DNA molecules stretched between the surface of a glass slide and a magnetic bead (labelled respectively bead 1 and bead 2) is monitored, in the presence of Isw1a (but without ATP). (ii) Typical image of these two particular beads. (iii) While the force is controlled (upper continuous trace), the resulting extension of the molecules is recorded. At F = 1.7 pN (trace (1)) and F = 1.0 pN (trace (2)), the two beads show restricted Brownian fluctuations about a mean value that varies with the stretching force. At 0.7 pN (trace (3)), while the length of the DNA bound to bead 2 is unchanged (except for a quick transient decrease at ∼430 sec blown up in the inset), the DNA anchored to bead 1 exhibits a rapid decrease in extension. (B) Representative TEM observations of a 4 kb linear DNA in absence (i) or presence of Isw1a (ii) without ATP. The reaction was carried out in 20 mM Tris-HCl pH 8, 30 mM KCl, 17.5 mM NaCl, 1 mM Hepes pH 7.6, 0.75% glycerol with 750 nM of DNA (in bps, ie 0.19 nM in molecule) and 20 nM of Isw1a for 20 min at 30°C. DNA collapse is observed on some DNA molecules in presence of Isw1a, while naked DNA molecules are still present. Scarce punctual binding (top right molecule) can also be seen (red arrow). Scale bar represents 200 nm.

In order to study the binding of Isw1a to a single DNA molecule, we used a magnetic trap [24] to exert a controlled force on DNA. Briefly a 3.6 kbp DNA fragment was tethered at one end to a glass slide and at the other end to a magnetic bead (with a diameter of 1 µm). Magnets were then used to set the force (F) stretching the molecule and to control the rotation of the bead. Video microscopy was used to monitor the position of the bead and provide a real-time measurement of the distance of the bead to the glass surface (see Figure 1A panel i). In parallel, the TEM analysis of complexes formed by Isw1a on various DNA substrates (supercoiled plasmids, linear DNA, and nucleosomal substrates) provide information on the binding mode and conformational changes induced by Isw1a on naked and chromatinized DNA.

### Isw1a binds cooperatively to DNA molecules in an ATP independent manner

With the magnetic trap set-up we can simultaneously monitor the change in extension of up to 50 beads tethered to the surface. Figure 1A panel ii shows a typical image of two such beads, labelled Bead1 and Bead2. At rather high forces (>1 pN), the beads undergo restricted Brownian fluctuations whose vertical amplitude is given by the stiffness of the anchoring DNA molecule (Figure 1A panel iii). In the presence of Isw1a (at a typical concentration of 0.5 nM), we observe below a force of F∼0.7 pN a very sharp reduction in the distance of Bead1 to the surface (the tethering DNA length decreases by 360 nm in 1 s and by a further 70 nm in the next 3 min.), while no such changes are observed on the nearby Bead2 anchored by exactly the same kind of DNA molecule (Figure 1A panel iii).

These observations are coherent with bulk experiments that have shown that Isw1a can bind to short DNA fragments in the absence of ATP [32], [33]. The observed behaviour is however typical of highly cooperative binding (and first order transitions) which necessitates the nucleation of a critical number of protein/DNA complexes which then proceed to cover the molecule by recruiting more proteins [34]. Notice also the large increase in the extension fluctuations in the presence of protein complexes, which implies that the interaction of the complex with DNA is very dynamic. This picture is essentially unaffected by repeating the experiment in the presence of ATP. As shown in Figure S2A, at 0.7 pN, dynamic cooperative binding of Isw1a to DNA is similarly observed both with and without ATP. This result could be related to previous papers reporting the cooperative activity of ISWI family remodelling enzymes [35], [36], [37].

To check that our interpretation was correct, we used transmission electron microscopy (TEM) to study the binding of Isw1a complexes to a linear blunt DNA of roughly similar length. In agreement with the magnetic trap experiments, we observed an ATP-independent cooperative binding of Isw1a on 4 kbps linear substrate. While naked DNA molecules could be seen at these protein and salt concentrations, we observed frequent collapses of linear molecules consistent with the cooperative shortening of DNA observed in the magnetic trap experiments (Figure 1B panel ii). This shortening (see Figure 1B panel ii) is due to intra- or inter-molecular bridging (see Figure S2B) that could be mediated by DNA-protein-protein interactions or by the ability of Isw1a to associate two DNA strands facilitating the subsequent binding of other proteins.

### Observing single Isw1a binding/unbinding events

The binding cooperativity of Isw1a and its dynamic nature present an experimental challenge to a detailed investigation of the interaction of the protein with DNA: it increases the experimental noise and does not allow one to precisely determine the number of complexes present on DNA. Fortunately, increasing the tension on DNA reduces the affinity of Isw1a, possibly by increasing the energy of looped DNA and reducing the probability of bridging between nearby DNA segments. As shown in Figure S3A, at high forces the DNA's extension is unchanged even in presence of Isw1a. At lower forces (0.75 pN), Isw1a binds cooperatively to DNA, which results in a rapid decrease in the molecule's extension. Increasing the force back to values above 1 pN unravels the complexes and restores the DNA to its full extension.

**Figure 2:**
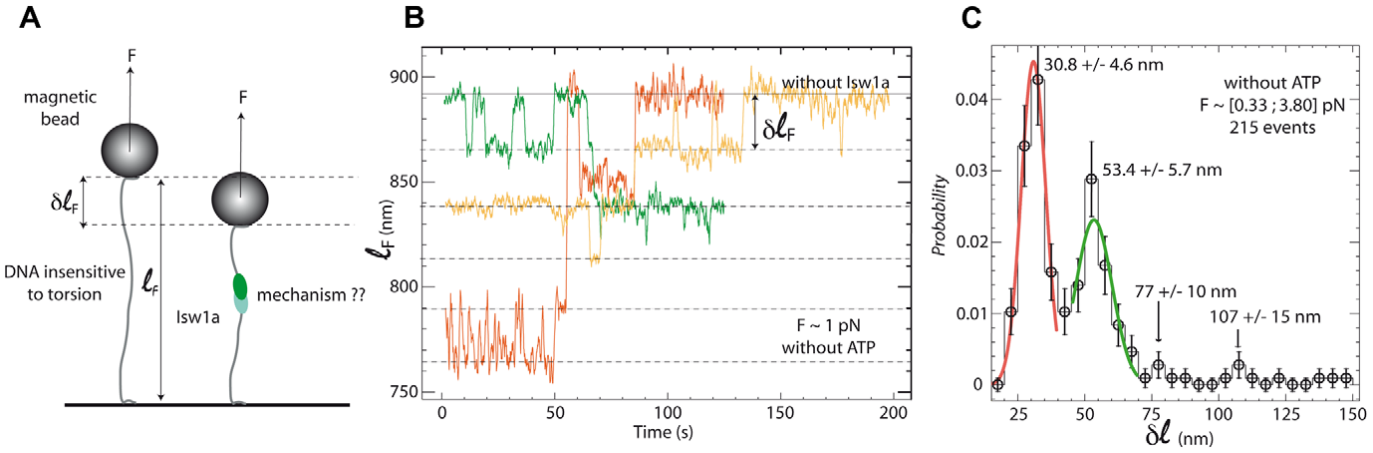
DNA variations in length induced by Isw1a binding and unbinding. (A) Schematic drawing of the experiment monitoring individual Isw1a binding events. (B) Recordings (averaged over 0.5 s) of the extension of three nicked DNA molecules of extension ℓ_F_∼1.2 µm, in the presence of Isw1a and without ATP. Successive increases (or shortenings) of the extension are observed as the force is increased (decreased) above (below) ∼1 pN, each of them being a multiple of δℓ = δℓ_F_/λ_W LC_∼30 nm. These variations in length are attributed to the binding or the unbinding of one or more Isw1a complexes. (C) Size distribution of δℓ for 215 events, for forces comprised between 0.33 and 3.8 pN.

Thus by modulating the tension on the DNA one can control the binding affinity of Isw1a and sometimes observe discrete decreases (or increases) in the DNA's extension corresponding to one (or a few) complexes associating with (or dissociating from) the molecule (Figure 2). The histogram of these discrete changes in extension exhibits four distinct peaks at multiples of δ = 28±2 nm. We therefore conclude that the cooperative association of Isw1a along the DNA molecule reduces its extension by about 28 nm (Figures 2B and C). At a force F∼1 pN, we rarely observed a telegraphic-like signal in the time traces of the DNA's extension that corresponds to single enzymatic complexes binding and unbinding from the molecule (Figure S3B). These rare nucleation events probably associated to bridging of DNA segments in a loop have a characteristic size δ_i_ = 30.8±4.6 nm: The distribution of on and off times (τ_on_, τ_off_) is exponential with mean values: <τ_on_> = 64 s and <τ_off_> = 69 s. The off-time is typical of diffusion-limited binding rates at the ∼1 nM concentration of enzyme used here. The on-time suggest that the binding affinity of the nucleation complex at the force studied is ∼1 nM, which implies that at zero force its binding affinity is ∼1 pM (see Data S1).

### Isw1a binding cooperativity is favored by the proximity of 2 DNA strands

In order to get insight on the factors affecting cooperative binding of Isw1a onto DNA, we studied by TEM the effect of Isw1a concentration upon its binding on two types of DNA substrates: linear and negatively (−) supercoiled (sc) (see panels A and B on Figure S4). At a concentration of 50 nM, corresponding to a molar ratio of 1 protein per 15 bps, up to 80% of DNA molecules were complexed to Isw1a (Figures S4A panel iv and S4B panel iv), with 35% of Isw1a complexes bridging a distance greater than 50% of total DNA length. At lower protein concentrations of 10 and 2 nM (protein/bp ratios of 1∶75 and 1∶375), we observed free DNA (53 and 82% respectively) coexisting with molecules coated on half of their length with a large amount of proteins (1.5 and <0.5% respectively) (Figure S4A panels ii and iii, red arrows). This is in agreement with the magnetic trap experiment and with the cooperative binding mode of the protein. Interestingly, it seems that the proximity of two DNA strands in scDNA favors the cooperative binding of Isw1a complexes (Figures S4A panels ii–iii compared to S4B panels ii–iii) as the scDNA molecules appeared zipped and thickened by the increasing amounts of protein complexes present along them. On a mixture of linear and (−) scDNA molecules (Figure S4C), the latter were more frequently coated by Isw1a than the former (6% of unbound scDNA vs 25% of bare linear DNA at 50 nM Isw1a). This was further confirmed by quantification in a gel shift assay (see Figure S5). We thus conclude from both TEM observations and single molecule measurements that the Isw1a complex assembles on DNA through a zipper-like bridging of nearby segments. This assembly could result from direct protein-protein interactions or from an indirect mode where the bridging of DNA by one (or a few) complexe increases the probability of bridging of proximal segments on DNA by further complexes.

**Figure 3:**
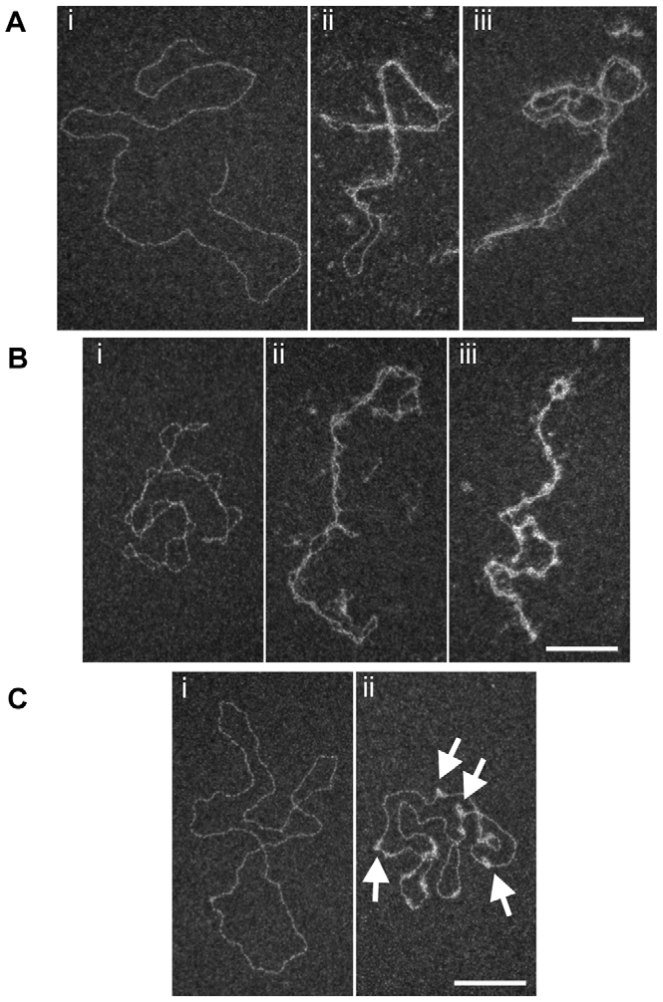
Binding of Isw1a on linear and negatively supercoiled DNA. (A) Cooperative binding and bridging of Isw1a along DNA molecules on linear DNA at 140000× magnification. Control DNA (i), reacted with Isw1a 50 nM (ii) and Isw1a 250 nM followed by gel filtration purification (iii) are presented here. (B) Binding and bridging of Isw1a along DNA molecules (−) scDNA at 140000× magnifications. Control DNAs (i), reacted with Isw1a 50 nM (ii) and Isw1a 250 nM followed by gel filtration purification (iii) are presented here. (C) Panels (i) and (ii) corresponds to the 50-fold dilution in pure H_2_O of 0 or 50 nM Isw1a binding on (−) scDNA presented in Figures 3B (a) and 3B (b), respectively. White arrows show Isw1a binding at apexes. For all images, scale bars represent 100 nm.

At high magnification, we could observe some 4 kb linear DNA molecules folded in two (Figures 3A panels ii–iii). This phenomenon is never observed on control DNA (Figures S4A panel i and 3A panel i). The presence of proteins is evidenced by the frequent observation of a fuzzy density between the two DNA-strands (Figure 3A panels ii–iii). Some circular forms where DNA is folded in two parts can also be seen (Figure S4A panel iv). On (−) scDNA (at a protein/bp ratio of 1∶15), proteins are clearly seen to bridge DNA crossovers (Figure 3B panel ii). In saturating conditions (proteins/bp ratio of 1∶3 - 250 nM for 750 nM in bps-and purification of the complexes) (see Figure 3B panel iii), proteins could even fill the gaps between both DNA strands. Interestingly, when stretching scDNA in a toroid form by large dilution in pure water, which increases charge repulsion along the DNA backbone [38] (Figure 3C panel i), Isw1a bridges were maintained mainly at apexes (Figure 3C panel ii, white arrows). This observation is consistent with the magnetic trap data that sometimes observes single binding/unbinding nucleation events associated with DNA looping (Figure S3B and Figure 3C panel ii).

### The formation of a Isw1a/DNA complex does not significantly affect the DNA twist

These TEM observations led us to investigate the effect of Isw1a binding on positively (+) and negatively (−) scDNA. Indeed, in many cases the binding of a protein complex to DNA affects its twist: overwinding [39] or underwinding it [40], [41]. Magnetic traps, which allow to twist DNA by simply rotating the pulling magnets, provide an easy assay to study that effect on single molecules.

**Figure 4:**
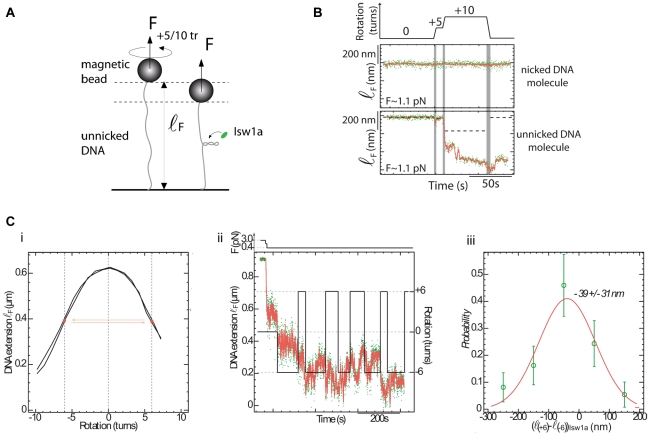
Effect of Isw1a binding on DNA twist. (A) Scheme of a torsion experiment when the DNA molecule is not nicked (no ATP). (B) Simultaneous recordings of the extension of two DNA molecules (one nicked and one not) in the presence of Isw1a (F = 1.1 pN) as a function of the number of rotations *n* of the pulling magnets (upper continuous trace). While the extension of the nicked DNA molecule (upper trace) remains constant as the magnets are rotated, the extension of the unnicked one (lower trace) decreases rapidly once it buckles to form supercoiled (i.e. when *n* = +10 turns). (C) (i) Extension-versus-rotation curve at 0.4 pN for an unnicked DNA molecule. (ii) Recording of the extension of the same molecule in the presence of Isw1a while imposing either +6 or −6 turns. The extension of the molecule (ℓ_F_ (±6) is reduced by a similar amount whether the molecule is rotated by +6 or −6 turns. (iii) Histogram of the mean difference in extension between successive ±6 turns (ℓ_F_ (+6)−ℓ_F_ (−6)) in the presence of Isw1a. The histogram is gaussianly distributed with a mean value of −39±31 nm.

First, we compared the binding of Isw1a on nicked DNA molecules (which are insensitive to torsion) to its binding on un-nicked positively coiled molecules (Figures 4A and 4B). At this force (F = 1.1 pN) and at zero twist no change in extension is observed in presence of Isw1a on either nicked or un-nicked molecules. Binding of the protein complex is not induced upon twisting the un-nicked molecule by +5 turns which twists the DNA but doesn't writhe it (the twist is below the bucking threshold). One needs to turn the molecule by +10 turns, generating supercoils on which the protein complex can nucleate to immediately see a sharp decrease in its extension due to the cooperative binding of Isw1a complexes (Figure 4B, bottom trace). Notice that the nicked molecule's extension is unaffected and doesn't bind the protein at this force. Upon reverting to zero twist, the supercoiled molecule did not revert to its initial (uncoiled) extension as it does in absence of proteins, but rather persisted at its shorter protein bound one, suggesting that the proteins complexes were bridging the plectonemic supercoiled structure, in agreement with the previous TEM observations.

Second, we monitored protein binding on (−) and (+) scDNA, to investigate whether protein binding was generating compensatory supercoils that affected differently the extension of (−) and (+) scDNA (Figure 4C panel i). However, the very dynamic and cooperative binding of the Isw1a complex, especially on scDNA, is complicating this assay by increasing the noise in extension measurements.

To overcome that problem we have measured the average difference between Isw1a complexes on a DNA molecule alternatively and rapidly supercoiled by ±6 turns (Figure 4C panel ii). Thus, any compensatory Δn supercoils generated by the complexes will be reflected in a consistent average difference between the change in extension of (±) scDNA. The histogram of the mean difference in extension between successive supercoiled states is a Gaussian distribution (Figure 4C panel iii), with mean: −39 nm and standard deviation: 31 nm. Since the size of a single plectoneme is 64 nm, this translates in a difference in the number of compensatory turns of: Δn = −0.30±0.24. Since the change in extension between bare DNA and the DNA bound with Isw1a (at zero rotation) was δℓ∼250 nm, and since the binding of a single complex reduces the extension by δ∼28 nm (see above), we conclude that there are about m = δℓ/δ∼9 complexes on this scDNA. Hence each complex generates only δn = Δn/m = −0.033±0.026 compensatory turns. Thus within our experimental error, we conclude that the binding of Isw1a to DNA while bridging the plectonemic structure does not affect its linking number.

### The Isw1a/DNA complex can bridge between two DNA molecules

**Figure 5:**
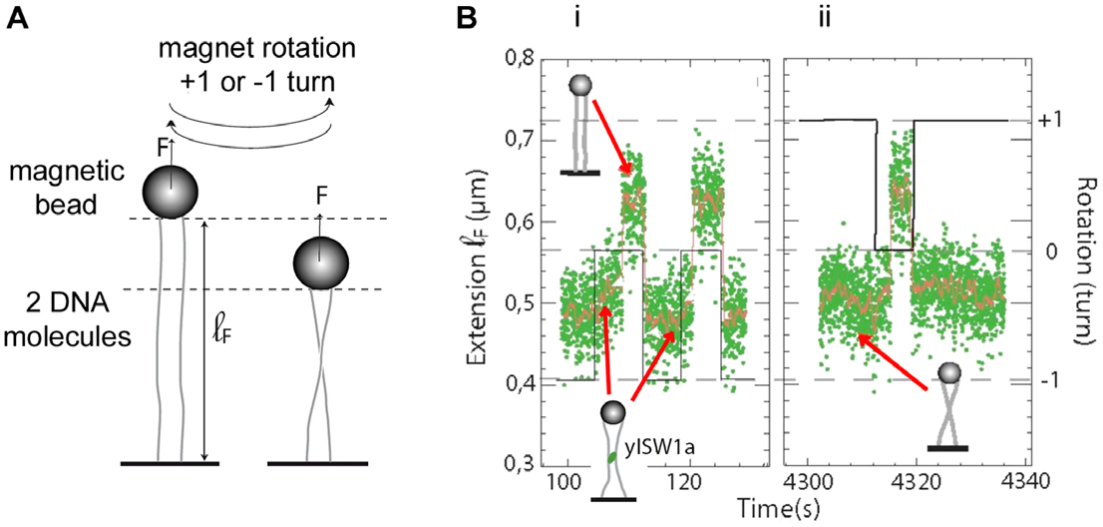
DNA bridging between two molecules. (A) Scheme of the experiments testing the binding in absence of ATP of Isw1a to two braided DNA molecules. (B) Recordings of the extension (dots) of two molecules braided by (i) −1 or (ii) +1 turn, in the presence of Isw1a at F = 0.9 pN. The continuous black line shows the amount of braiding (number of magnets rotations). The red curve corresponds to an average of the raw data over 0.25 s. Notice that in both cases following a return to zero rotation of the magnets (unbraiding of the DNA), the extension of the molecules increases back to its initial position albeit with a stochastic delay due to the transient bridging of the braid crossing by a single Isw1a molecule.

To confirm our TEM observations of DNA bridging by Isw1a, we braided by one turn two DNA molecules stretched at a force that prevented spontaneous assembly of Isw1a on the molecules (Figure 5A). We reasoned that if Isw1a interacts with a segment of DNA then it might interact with the opposite DNA segments in the crossover generated by rotating the magnets by ±1turn. Indeed this is what we observed. When braiding the molecules their extension decreases for simple geometric reasons (as in a twisted swing). However when unbraiding them in presence of Isw1a they do not immediately return to their full extension (as observed in absence of protein) but rather take a few seconds to snap back to their original extension (Figure 5B panels i–ii). As it was similarly argued for TopoII enzymes, this result shows that the complex bridges between the two DNAs at their crossovers [42].

### Isw1a bridges linker DNA on nucleosomal substrates

We have shown that the Isw1a has surprising ATP-independent ability to bind bare DNA dynamically and to bridge between distinct DNA molecules or between segments within one molecule. Hence, we further investigated how these binding properties could be implicated in remodeling activity on nucleosomal substrates.

We first reconstituted nucleosomal arrays on a negatively supercoiled plasmid of 5.4 kb. In our conditions [30] nucleosomal arrays contained on average between 10 and 30 nucleosomes per DNA molecules (Figure S6A panel i). Notice that as the reaction buffer contained magnesium salt, some of the chromatinized plasmids were quite condensed on TEM grids. When adding Isw1a to these substrates in presence of ATP, the nucleosomal arrays appeared even more condensed, but it was difficult to distinguish Isw1a binding on those structures (Figures S6A left panel ii). The presence of less saturated nucleosomal substrates in our reconstitution allowed us to observe the binding of Isw1a more specifically on linker DNA at the entry and the exit of the nucleosome (see red arrows on magnifications, Figure S6A right panel ii). As observed on bare DNA in absence of ATP, Isw1a was seen bridging together the DNA arms of the nucleosome, which turn up to be a preferential substrate for Isw1a loading.

### In presence of ATP the Isw1a cooperative complex efficiently remodels nucleosomes

**Figure 6:**
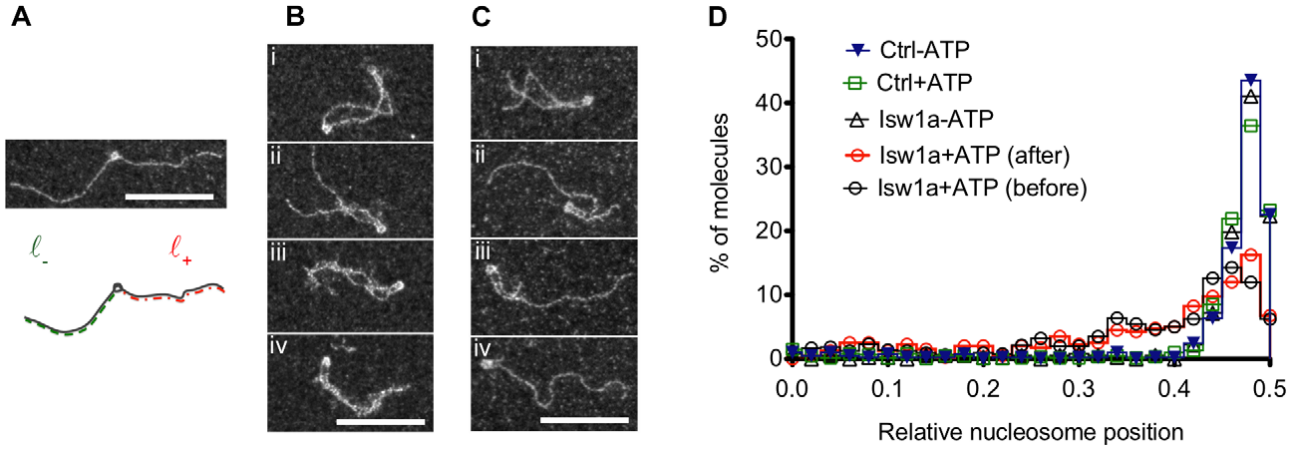
Isw1a remodeling activity on a single nucleosome. (A) Image of 845 bps mononucleosomal DNA containing a histone positioning 601 sequence and schematic drawing representing the lengths measured in the analysis: in the non-oriented experiment ℓ_−_ represents the shortest DNA arm, ℓ_+_ the longest. Representative images of mononucleosomes on 845 bps DNA with 10 nM of Isw1a in absence (B) or presence (C) of 75 µM of ATP. Scale bars represent 100 nm. (D) Relative nucleosome position probabilities calculated as the ratio of (ℓ_−_)/( ℓ_+_+ℓ_−_) for five different experimental conditions.

In order to get insight on the compatibility of this binding with remodeling activity of Isw1a in TEM conditions, we pursued our investigations with a single nucleosome reconstituted on a 601 nucleosomal positioning sequence flanked by two relatively long DNA arms of 360 and 338 bps leading to exiting nucleosomal arms of 120 and 112 nm respectively (with a relative nucleosome position at 0.48) (Figure 6A). Our various reconstitutions on those rather long substrates led to a majority of mononucleosomal arrays (greater than 50%), but bare DNA (up to 30%) or polynucleosomal substrates (up to 20%) could also be seen. We focused our analysis on single nucleosomal templates. These are shown in Figure S6B after incubation at 30°C with or without ATP (panels i and ii respectively, no Isw1a). Careful length measurements of the DNA shortest and longest arms on all the mononucleosomal species (Figure 6A) showed an average positioning of nucleosome at the expected relative position of 0.48 with 80% of the nucleosomes observed between relative positions 0.43 and 0.50 (Figure 6D, Figures S7A and B, green and blue).

In presence of Isw1a bridging of the DNA arms was observed (Figures 6B and 6C) and Figures S6B panels iii–v), as on polynucleosomal arrays. The nucleosome was usually seen at the apex of the structure, though bridging by Isw1a was not always observed in the nucleosome vicinity but also at DNA crossovers (Figure 6B). The fuzzy aspect of the protein-DNA complexes allowed us to measure the length of the DNA arms in presence of Isw1a on most of the single nucleosome molecules, except in some large entangled DNA complexes (data not shown).

First, at our measurement resolution (5 nm), we did not observe any significant change in the length of the DNA arms in presence or absence of Isw1a and/or ATP when summing the lengths ℓ_−_ and ℓ_+_ of the flanking regions of the nucleosome (Figure S7, Y axis), the median value being at 232 to 235 nm. This is in agreement with the bridging property of Isw1a and stand in contrast to the reported large loops formed by the SWI/SNF and RSC complexes [18], [20], [22], [43].

Second, as expected, in absence of ATP Isw1a bridging does not remodel the nucleosome: its median relative position remaining in absence of ATP at 0.48 (percentile 10–90%: 0.43–0.50) as in the control (no Isw1a).

However, in presence of ATP (added before or after the Isw1a complexes had assembled cooperatively on the nucleosomal substrates), while bridging was still observed (83% of bridged molecules at 10 nM Isw1a and 40% at 1 nM before ATP addition), it did not prevent the remodeling activity of Isw1a. As shown on Figure 6D (open red (after) and black (before) circles), a clear repositioning of the nucleosome as compared to the control was observed for a substantial part of the population (median position 0.42 (percentile 10–90%: 0.11–0.48)). In presence of ATP 49% of the molecules have a nucleosome relatively positioned at less than 0.40 (56 pbs displacement or more) compared to only 8% in absence of ATP. At a lower Isw1a concentration (1 nM) corresponding to a molar ratio of ∼2∶1 Isw1a per nucleosome, remodeling of smaller extent is observed (Figure S6C panel iii), as only 22% of molecules have a nucleosome relatively positioned at less than 0.40. As the extent of remodeling seems to be correlated to the number of bridged DNA molecule before ATP addition, we conclude that nucleosome remodeling by Isw1a is not only unhindered by the cooperative complex bridging the two DNA arms flanking the nucleosome, it may even be facilitated by its presence.

On these large substrates, where the influence of the molecule's extremities is weaker than in the smaller substrates usually studied [15], Isw1a was able to reposition a nucleosome by more than 120 bps (25% and 11% of the molecules at 10 nM and 1 nM Isw1a respectively). This remodeling away from the center was observed on initially centrally positioned nucleosome. The broadening of the probability distribution of nucleosome positions results probably from the stochastic non-directional remodeling activity of the enzyme.

To further test whether the Isw1a remodeling activity had a preferred direction or was random, we studied it on nucleosomal substrates that were dimerized by a streptavidin bridging their biotin labeled extremity. As shown on Figure S8A panel i, we can discriminate in this dimer between the nucleosomal flanking regions ℓ_1_ (from the nucleosome to the free DNA end) and ℓ_2_ (between nucleosome and streptavidin). The analysis of nucleosome positions (Figures S8B–F), led us to the conclusion that the nucleosomes were repositioned equally on both sides of the central position indicating that Isw1a doesn't translocate the nucleosome in a preferred direction.

These results show that repositioning of nucleosomes is efficient in presence of ATP under conditions in which cooperative binding of Isw1a is observed and where one might have expected the cooperative complex to hinder repositioning. The dynamic bridging of linker DNA at the entry/exit sites of the nucleosome could in fact be a means for Isw1a to be recruited at the nucleosomal substrate, regulating and facilitating its active remodeling.

## Discussion

The yeast chromatin remodeling complex Isw1a, member of the ISWI family [44], has been shown to move nucleosomes *in vitro* [15] and to act as a gene repressor *in vivo* [45]. To better characterize its mechanism, we investigated its interaction with bare DNA molecules and reconstituted nucleosome particles in single molecule magnetic trap experiments and TEM imaging. We have seen that at low forces its interaction with DNA is highly cooperative and very dynamic (with or without ATP). This dynamic behavior, evidenced in the increased fluctuations in the molecule's extension, is probably due to the kinetics of protein-protein and protein-DNA interactions. By increasing the ionic concentration and varying the force the affinity of the complex can be reduced to such an extent that the successive assembly (or disassembly) of one (or a few) complexes can be observed. Notice that similar results were obtained with another remodeling factor, CHD1, belonging to the NURD/Mi-2/CHD family (Figures S3C).

**Figure 7:**
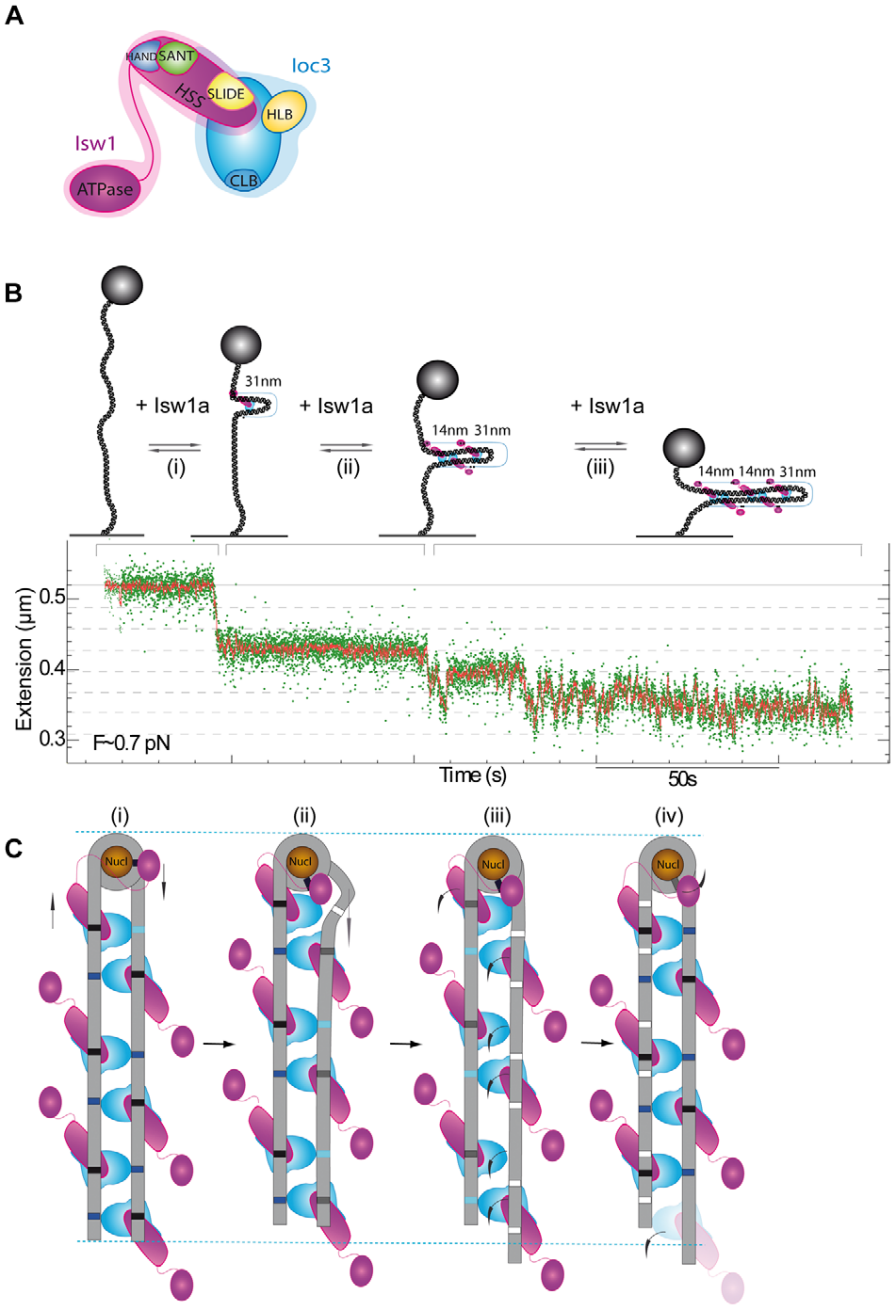
Possible model for the formation of an Isw1a fiber along bare and nucleosomal DNA in magnetic tweezers and TEM experiments. (A) Schematic structure of Isw1a according to ref [46]. The protein chain connecting HAND and ATPase domain is flexible, which may contributes to chromatin remodelling. (B) The formation of the Isw1a fiber on stretched DNA could proceed in successive steps: (i) binding/unbinding of a single nucleating complex which grows by binding of additional complexes ending in a zipped-up DNA molecule (ii–iii). A typical time trace taken at 0.7 pN of the extension of a DNA molecule in presence of Isw1a (without ATP) is shown. The dashed lines correspond to the change in extension upon addition of a single complex deduced from the measurements shown in Figure 2. (C) On nucleosomal substrate, the nucleation of Isw1a (i) may proceed from the vicinity of the nucleosome where the two DNA flanking regions provide an adequate substrate for the nucleation of a Isw1a fiber that proceeds by bridging the two arms of the nucleosomal substrate. (ii–iv) In presence of ATP, the Isw1a protein close to the nucleosome generates a DNA deformation, which may weaken the Ioc3 and Isw1 interaction with DNA. Then, the other Isw1a units along the DNA zipper act as “wheels” that in presence of ATP translocate the DNA molecule as a chain in a conveyor belt. As the nucleosome is remodelled, one DNA arm length is decreasing and some of the Isw1a bridges are weakened resulting in fewer bridged molecules after remodelling. Dark blue and black sites represent strong DNA binding of Ioc3 and Isw1, respectively. Light blue and gray sites represent weak binding sites of Ioc3 and Isw1, respectively, allowing the propagation of the DNA deformation. White sites show previously linked sites and released by the different sub-domains of Isw1a.

The crystal structure of Isw1a (lacking its ATPase subunit) bound to two 48 bps DNA fragments has recently been published [46]. The HAND, SANT and SLIDE (HSS) subdomains of Isw1 (lacking its N-terminal ATPase sub-domain) interact with one fragment while the coil-linker-binding (CLB) domain of the globular core domain Ioc3 binds to a second fragment (Figure 7A). On the long stretched DNA used here, this interaction results in a bending of the intermediate fragment (between the two binding site) and a subsequent reduction in the molecule's extension of 31 nm. This value is determined by the minimization of the energy required to bend the DNA against the stretching force (29): the larger the bending radius the smaller the bending energy, but the larger the work against the stretching force. As observed in our experiments, on a supercoiled DNA molecule the presence of nearby fragments favours the binding of a Isw1a complex without having to pay the energetic price of bending the DNA against the tension in the molecule. Hence formation of a complex with Isw1a is immediate once a molecule forms supercoils, (see Figure 5 B).

Like the structural investigation cited above, most investigations of Isw1a look at its remodelling activity on nucleosomes flanked by short DNA fragments. Consequently these investigations could not observe the cooperative assembly of Isw1a around the nucleation point provided by the binding of the initial complex. This cooperative assembly by bridging opposite DNA fragments is observed to reduce the DNA's extension by δ∼28 nm. The size of the bridged fragments is thus about 50 bps which could to correspond to a dimer of Isw1a in opposite orientation (as in Figure 4d of ref. [46]).

This possibility of Isw1a to act in trans (to bridge between different DNA molecules) and to act cooperatively is unexpected and reminiscent of the action of H-NS bridges in the organization of bacterial chromatin [47]. Indeed, bridging mechanisms were first described for proteins involved in the organization and regulation of bacterial genomes such as H-NS or LrpC [31], [47], [48]. Adhesion energies brought into play for each bridge are probably quite low (1 k_B_T for H-NS [48]). Successive associations of these bridges increase the adhesion while maintaining a highly dynamic state within a zipper–like structure. We have shown here that Isw1a binds cooperatively onto DNA by DNA-protein bridging and polymerisation along the two DNA segments. TEM images show that in absence of ATP the assembly of Isw1a complexes along DNA is able to fold the molecule in half thus bringing its extremities in close proximity. This observation suggests that the dynamic assembly of the protein complexes proceeds such as to maximize its adhesion energy with DNA. Notice that the Isw1a cooperative assembly is not sufficient by itself to remodel the nucleosome in the absence of ATP. Indeed, Isw1p has been shown to directly interact with the histones H3 and H4 [49] and the Isw1a ATPase activity is stimulated by nucleosomes [32], [50]. However binding of Isw1a is largely increased by the presence of extranucleosomal DNA at both entry and exit sites [50]. Hence, we suggest that the DNA bridging properties of Isw1a allow for its recruitment at nucleosomal substrates. Then in the presence of ATP, effective remodeling can proceed on the bridged extranucleosomal DNA structures.

The study recently published [46] of dinucleosomal substrates bound by short (25 bps) DNA-linkers suggested a model whereby the remodeler acts as a “protein ruler” that sets the distance between the nucleosomes by reeling in one nucleosome towards the other. Our studies investigated the interaction of Isw1a with much longer DNA fragments. Both single molecule experiments and TEM observations show that binding of Isw1a on DNA is cooperative and ATP independent. On a nucleosomal substrate the protein complex is initiated by binding to the two nearby DNA fragments at the entry and exit sites of a single nucleosome. From that nucleation point assembly of a Isw1a fiber is progressing cooperatively and zipper-like by steps of about 14 nm (∼50 bps) compatible with binding of a Isw1a dimer (in opposite orientation) to the two adjacent DNA strands, see Figure 7B. This model is supported by TEM imaging on bare DNA molecules (see Figure 6B panels i–iv). It generalizes to longer DNA linkers the idea of Isw1a acting as a “protein ruler”, but with ticks roughly separated by 50 bps.

In presence of ATP, the Isw1a DNA dynamic bridging activity discovered here suggest that this assembly could play the role, as suggested by the recent structural data [46], of a “protein ruler” albeit with as many ticks as there are complexes in the assembly. Indeed, the TEM observations suggest that contrary to naïve expectations the cooperative assembly of Isw1a along DNA appears to assist remodelling.

Our single molecule studies and TEM observations suggest a possible “conveyor belt” model for the mechanism of Isw1a remodeling of nucleosomal DNA, see Figure 7C. In this model the various Isw1a units along the DNA zipper act as “wheels” that in presence of ATP translocate the molecule as a chain in a conveyor belt. The recent structure of Isw1a indeed point to some flexibility either in the DNA linker or the protein domain between the HAND and ATPase domain that could mediate the translocation of DNA segments between nearby units. Depending on their degree of mechanical coupling subsequent units may work synchronously to translocate the molecule [51] or asynchronously using the aforementioned inherent flexibility to reduce tensions during translocation. This configuration could allow the local isolation of a DNA domain while allowing the active complex to interact with and displace the nucleosome. The bridging of DNA, which is a dynamic process, could thus assist or regulate the relative positioning of nucleosomes.

## Supporting Information

Figure S1 **Control of Isw1a quality.** Samples were electrophoresed on a NuPAGE 4–12% Bis-Tris gel (Invitrogen) as per manufacturers instructions. The gel was stained with Instant Blue (Expedeon protein Solutions) for visualisation of protein bands.
(TIF)

Figure S2 **Isw1a binding with and without ATP.** (A) Typical shortenings of the length l_F_ of two DNA molecules of 1.2 µm, stretched at 0.7 pN, in the presence of Isw1a, without ATP (upper trace) or with 100 µM ATP (lower trace). No significant difference is observed with or without ATP. (B) Large complexes containing several DNA molecules aggregated together with many Isw1a complexes could also be seen in TEM imaging. Binding conditions: 750 nM in bp (4 kb linear DNA)+20 nM Isw1a in binding buffer without ATP during 20 minutes at 30°C.
(TIF)

Figure S3 **Modulation of the cooperativity with force.** (A) Recording of the end-to-end extension of a DNA molecule of 1.2 µm, in the presence of Isw1a without ATP: at low force (0.75 pN), several complexes of Isw1a bind dynamically to DNA reducing significantly its extension. Pulling at high force (3.8 pN) forces the complexes to unbind sequentially from the molecule, which recovers its full extension. Two successive repeats of this procedure are displayed. (B) Distribution of on and off times at F∼1 pN. (i) Extension of a DNA molecule in the presence of Isw1a, without ATP, at constant force (1.0 pN). Raw data are in green, data averaged over 0.5 s appear in red. A telegraphic-like signal is observed, the DNA length oscillating between two values distant by 25.8±0.3 nm. (ii) Time distribution of τ_on_ and τ_off_ corresponding to the situation presented in (a): τ_on_ and τ_off_ are exponentially distributed and their mean values are respectively <τ_on_> = 63.9±26.8 s (over 45 events) and <τ_off_> = 69.1±18.6 s (over 44 events). (C) CHD1 behaves similarly to Isw1a on bare DNA. (i) Recording of the end-to-end extension of a DNA molecule in the presence of CHD1, without ATP, at 0.8 pN, in the following buffer: 10 mM Hepes pH 7.3, 50 mM KCl, 3 mM MgCl_2_, 0.1 mM DTT and 0.2% BSA. The extension of the molecule decreases rapidly due to the cooperative binding of multiple CHD1 complexes until the bead reaches the surface of the capillary and remains stuck on it (see the decrease in the amplitude of the brownian motion of the bead). (ii) In some conditions, one can record isolated binding/unbinding events as shown in this picture: the binding of one CHD1 complex decreases the DNA extension by 40±1 nm, at 0.5 pN. Its binding to DNA is very cooperative. By playing on force, one can isolate individual binding/unbinding events in the absence of ATP. The presence of ATP does not modify these observations.
(TIF)

Figure S4 **Binding of Isw1a on linear and negatively supercoiled DNA by TEM imaging.** Concentration range of Isw1a (0; 2; 10; 50 nM in panels i, ii, iii and iv, respectively) on linear (A), (−) scDNA (B) and a (1∶1) mixture of linear and (−) scDNA (C) (total DNA concentration: 750 nM in bp). Red arrows show Isw1a binding. Scale bars represent 200 nm. By analyzing the population of molecules on the TEM grids (n>800), we conclude that (A) bare linear DNA molecules represent 82% for 2 nM Isw1a, 53% for 10 nM Isw1a, and 20% for 50 nM of Isw1a, whereas linear molecules bridged by Isw1a on half of their length or more represent respectively 35%, 1.5% and less than 0.5% at 50, 10 and 2 nM Isw1a; (B) bare scDNA molecule represent respectively 10%, 37% and 57% at Isw1a concentration of 50, 10 and 2 nM, whereas molecules bridged by Isw1a on half of their length or more represent respectively 55%, 7% and 2% at 50, 10 and 2 nM Isw1a; (C) preferential binding of scDNA by Isw1a is observed: on a mixture of both DNA molecules, unbound linear DNA molecules represent 25%, 73% and 90% respectively at 50, 10 and 2 nM Isw1a as compared to 6%, 33% and 59% for scDNA. This binding preference is confirmed by gel shift assay (Figure S5).
(TIF)

Figure S5 **Binding of Isw1a on mixture of linear and supercoiled DNA by gel shift assay.** (A) Gel shift experiment of Isw1a binding (0–2–5–10–20 and 50 nM) 20 min at 30°C in binding buffer (20 mM Tris-HCl pH 8.0, 30 mM KCl) on a mixture of (−)-supercoiled DNA pBR322 (375 nM in bp) and 845 pb linear DNA (containing 601 positioning sequence for nucleosome) (375 nM in bp). 10% of sucrose was added just before loading (15 µL) on a 1% Agarose Gel in 0.5× TBE buffer and migration at 4°C. Gel was revealed by SyBR gold staining (Invitrogen) and imaged with Storm 840 apparatus (GE Healthcare). (B) Quantification of relative unbound DNA on 3 gel-shift experiments using ImageQuant 5.2 software (GE-Healthcare). Dots and dotted line (pBR322); squares and dash line (845 pb linear DNA).
(TIF)

Figure S6 **TEM analysis of Isw1a binding on chromatinized plasmid DNA.** (A) (i) Representative image of phiX174-RFI plasmid (750 nM in bp) (New England Biolabs) chromatinized with nucleosomes from calf thymus core particles in binding buffer with 100 µM ATP and 2 mM MgOAc for 20 minutes at 30°C without (left) and with 10 nM Isw1a (right panels). Bar represents 100 nm. (ii) Cooperative binding of Isw1a on nucleosomal arrays tend to compact chromatinized DNA and to bridge arms (red arrows) at the entrance and exit of the nucleosomes. White arrows show nucleosomes that can be clearly identified at apexes. (B) Representative images of mononucleosomes on 845 bps DNA containing a histone positioning 601 sequence: in absence (i, ii) or presence (iii–v) of Isw1a (1 nM (iii) or 10 nM (iv, v)), and in absence (i, iii, iv) or presence (ii, v) of 75 µM ATP. Binding or Isw1a is observed after 10 min incubation at 30°C (iv, right panel) and over 30 min incubation (iv, left panel). Scale bar represents 100 nm. (C) Effect of Isw1a concentration on remodeling efficiency. Nucleosome position on 845-bps 601 mononucleosomal substrates upon remodeling by Isw1a at 10 nM, 1 nM or in control protein buffer in 20 mM Tris-HCl pH 8.0, 30 mM KCl, 2 mM Mg(OAc)_2_ and ATP (75 µM) for 20 minutes at 30°C. (i) Relative nucleosome position probabilities calculated as the ratio of (*l*_−_)/(*l*_+_+*l*_−_). (ii, iii, iv) Dot plots of total length of extranucleosomal DNA (*l*_+_+*l*_−_) as a function of the difference of length between the longest and shortest DNA arm (*l*_+_−*l*_−_): (ii) control buffer+ATP (open green squares); (iii) Isw1a 1 nM+ATP (open black dots); (iv) Isw1a 10 nM+ATP (open red dots). Dotted grey lines represent median for each measure. Grey rectangles represent 10–90 percentiles. *n* is the number of molecules analyzed as a result of 2 independent experiments.
(TIF)

Figure S7 **Plots of total length (ℓ_+_+ℓ_−_) as a function of the difference between the longest and the shortest DNA arms (ℓ_+_−ℓ_−_).** (A) Control without ATP (blue triangles); (B) control with ATP (open green squares); (C) ATP added after Isw1a binding for 10 min (see Figure S6B (iv, right)) (open black circles); (D) ATP added before Isw1a (open red dots); (E) Isw1a without ATP (open black triangles); Dotted grey lines represent median for each measure. Grey rectangles represent 10–90 percentiles. *n* is the number of molecules analyzed as a result of 2 (graph C (ATP after Isw1a)), 3 (graphs E and A) or 5 (graphs D (ATP before Isw1a) and B) independent experiments.
(TIF)

Figure S8 **Binding to a dimer of biotinylated-845 bps mononucleosomal DNAs and remodeling activity of Isw1a.** (A) Image of the two biotinylated-845 bps mononucleosomal DNAs (containing a histone positioning 601 sequence) dimerized by streptavidin (for molecule orientation) and schematic drawing representing the lengths measured in the analysis: in this oriented experiment ℓ_1_ represent the length from the nucleosome to the free end, ℓ_2_ the length from the biotinylated end to the nucleosome (i). Representative images of mononucleosomes on 845 bps DNA with 10 nM of Isw1a with of 75 µM of ATP (ii). Scale bars represent 100 nm. (B–F) Quantifications of nucleosome positions on the dimer of 845 bps mononucleosomal DNA. (B) Relative nucleosome position probabilities calculated as the ratio of (ℓ_1_)/( ℓ_1_+ℓ_2_). (C–F) dot plots of total length (ℓ_2_+ℓ_1_) as a function of the difference between the longest and the shortest DNA arms (ℓ_2_−ℓ_1_). (C) (open blue triangles) control without ATP; (D) (open green squares) control with ATP; (E) (open black triangles) Isw1a without ATP; (F) (open red dots) ATP added before Isw1a. Dotted grey lines represent median for each measure. Grey rectangles represent 10–90 percentiles. *n* is the number of molecules analyzed.
(TIF)

Data S1 **Estimation of equilibrium constant k_D_.**
(DOCX)
