# Supplementary figures and images for: ATP-Independent Cooperative Binding of Yeast Isw1a to Bare and Nucleosomal DNA
Source: PLoS One. 2012 Feb 16;7(2):e31845. doi: 10.1371/journal.pone.0031845 (PMC3281020; doi:10.1371/journal.pone.0031845)

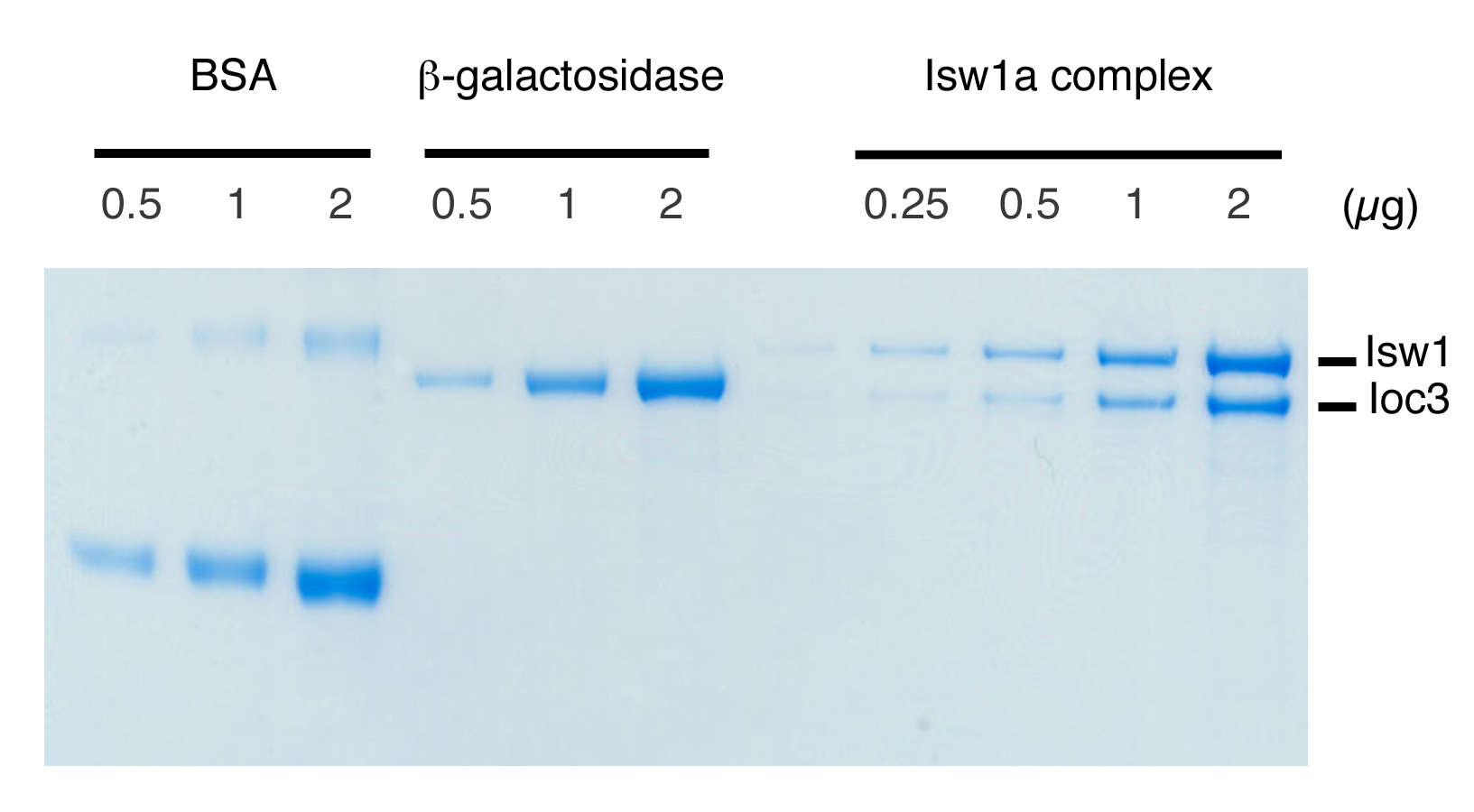

Supplement: Figure S1 — Control of Isw1a quality. Samples were electrophoresed on a NuPAGE 4–12% Bis-Tris gel (Invitrogen) as per manufacturers instructions. The gel was stained with Instant Blue (Expedeon protein Solutions) for visualisation of protein bands. (TIF) [file pone.0031845.s001.tif]

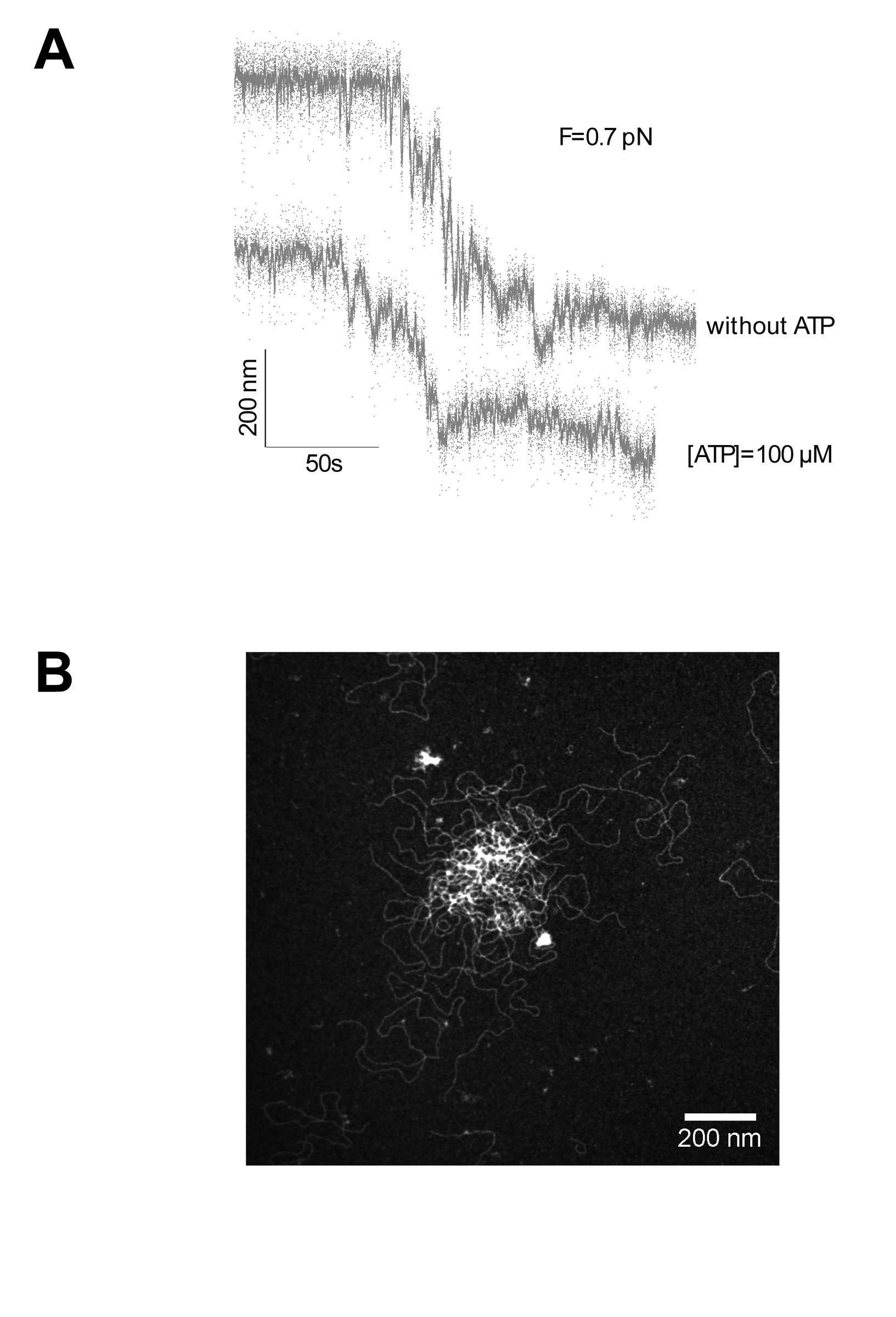

Supplement: Figure S2 — Isw1a binding with and without ATP. (A) Typical shortenings of the length lF of two DNA molecules of 1.2 µm, stretched at 0.7 pN, in the presence of Isw1a, without ATP (upper trace) or with 100 µM ATP (lower trace). No significant difference is observed with or without ATP. (B) Large complexes containing several DNA molecules aggregated together with many Isw1a complexes could also be seen in TEM imaging. Binding conditions: 750 nM in bp (4 kb linear DNA)+20 nM Isw1a in binding buffer without ATP during 20 minutes at 30°C. (TIF) [file pone.0031845.s002.tif]

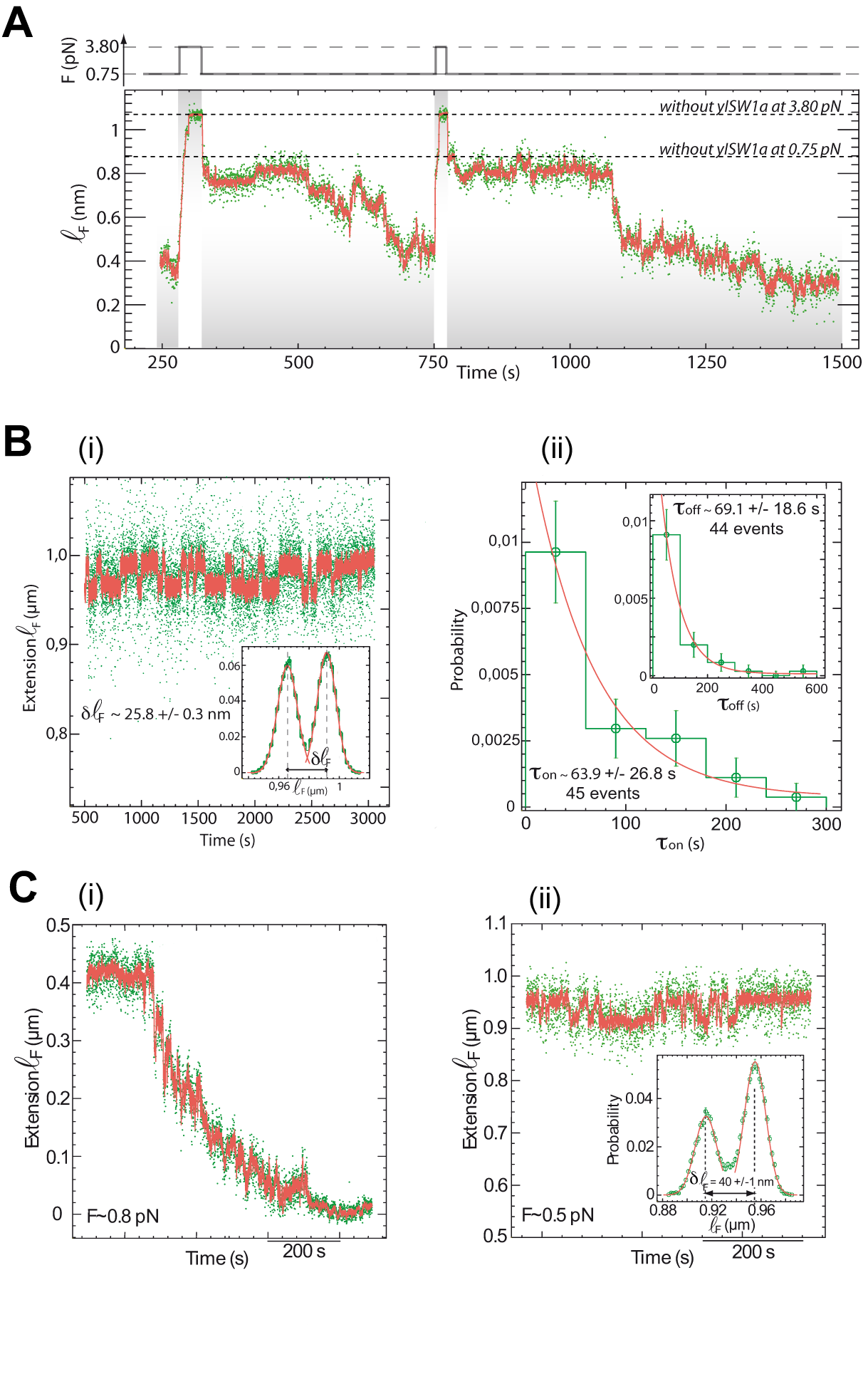

Supplement: Figure S3 — Modulation of the cooperativity with force. (A) Recording of the end-to-end extension of a DNA molecule of 1.2 µm, in the presence of Isw1a without ATP: at low force (0.75 pN), several complexes of Isw1a bind dynamically to DNA reducing significantly its extension. Pulling at high force (3.8 pN) forces the complexes to unbind sequentially from the molecule, which recovers its full extension. Two successive repeats of this procedure are displayed. (B) Distribution of on and off times at F∼1 pN. (i) Extension of a DNA molecule in the presence of Isw1a, without ATP, at constant force (1.0 pN). Raw data are in green, data averaged over 0.5 s appear in red. A telegraphic-like signal is observed, the DNA length oscillating between two values distant by 25.8±0.3 nm. (ii) Time distribution of τon and τoff corresponding to the situation presented in (a): τon and τoff are exponentially distributed and their mean values are respectively <τon> = 63.9±26.8 s (over 45 events) and <τoff> = 69.1±18.6 s (over 44 events). (C) CHD1 behaves similarly to Isw1a on bare DNA. (i) Recording of the end-to-end extension of a DNA molecule in the presence of CHD1, without ATP, at 0.8 pN, in the following buffer: 10 mM Hepes pH 7.3, 50 mM KCl, 3 mM MgCl2, 0.1 mM DTT and 0.2% BSA. The extension of the molecule decreases rapidly due to the cooperative binding of multiple CHD1 complexes until the bead reaches the surface of the capillary and remains stuck on it (see the decrease in the amplitude of the brownian motion of the bead). (ii) In some conditions, one can record isolated binding/unbinding events as shown in this picture: the binding of one CHD1 complex decreases the DNA extension by 40±1 nm, at 0.5 pN. Its binding to DNA is very cooperative. By playing on force, one can isolate individual binding/unbinding events in the absence of ATP. The presence of ATP does not modify these observations. (TIF) [file pone.0031845.s003.tif]

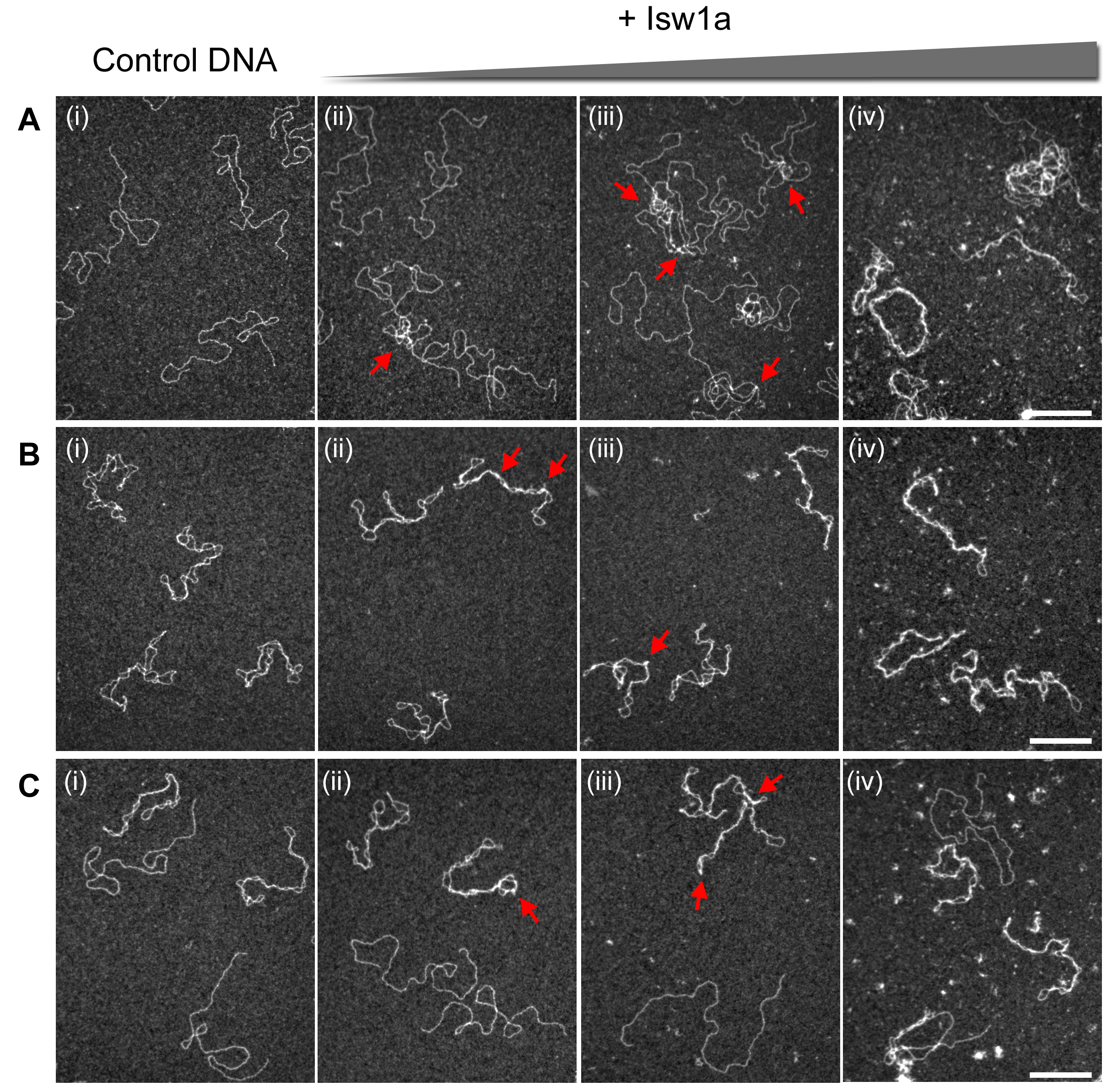

Supplement: Figure S4 — Binding of Isw1a on linear and negatively supercoiled DNA by TEM imaging. Concentration range of Isw1a (0; 2; 10; 50 nM in panels i, ii, iii and iv, respectively) on linear (A), (−) scDNA (B) and a (1∶1) mixture of linear and (−) scDNA (C) (total DNA concentration: 750 nM in bp). Red arrows show Isw1a binding. Scale bars represent 200 nm. By analyzing the population of molecules on the TEM grids (n>800), we conclude that (A) bare linear DNA molecules represent 82% for 2 nM Isw1a, 53% for 10 nM Isw1a, and 20% for 50 nM of Isw1a, whereas linear molecules bridged by Isw1a on half of their length or more represent respectively 35%, 1.5% and less than 0.5% at 50, 10 and 2 nM Isw1a; (B) bare scDNA molecule represent respectively 10%, 37% and 57% at Isw1a concentration of 50, 10 and 2 nM, whereas molecules bridged by Isw1a on half of their length or more represent respectively 55%, 7% and 2% at 50, 10 and 2 nM Isw1a; (C) preferential binding of scDNA by Isw1a is observed: on a mixture of both DNA molecules, unbound linear DNA molecules represent 25%, 73% and 90% respectively at 50, 10 and 2 nM Isw1a as compared to 6%, 33% and 59% for scDNA. This binding preference is confirmed by gel shift assay (Figure S5). (TIF) [file pone.0031845.s004.tif]

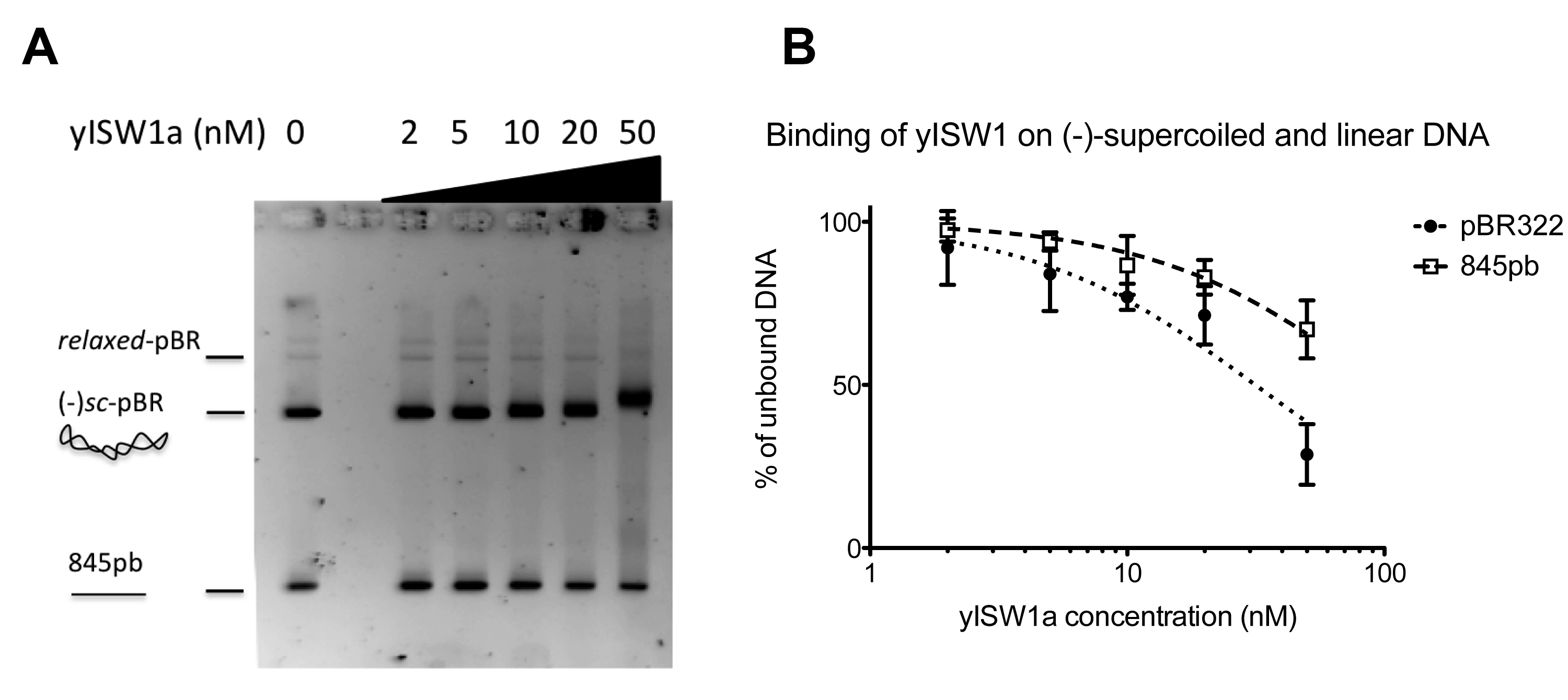

Supplement: Figure S5 — Binding of Isw1a on mixture of linear and supercoiled DNA by gel shift assay. (A) Gel shift experiment of Isw1a binding (0–2–5–10–20 and 50 nM) 20 min at 30°C in binding buffer (20 mM Tris-HCl pH 8.0, 30 mM KCl) on a mixture of (−)-supercoiled DNA pBR322 (375 nM in bp) and 845 pb linear DNA (containing 601 positioning sequence for nucleosome) (375 nM in bp). 10% of sucrose was added just before loading (15 µL) on a 1% Agarose Gel in 0.5× TBE buffer and migration at 4°C. Gel was revealed by SyBR gold staining (Invitrogen) and imaged with Storm 840 apparatus (GE Healthcare). (B) Quantification of relative unbound DNA on 3 gel-shift experiments using ImageQuant 5.2 software (GE-Healthcare). Dots and dotted line (pBR322); squares and dash line (845 pb linear DNA). (TIF) [file pone.0031845.s005.tif]

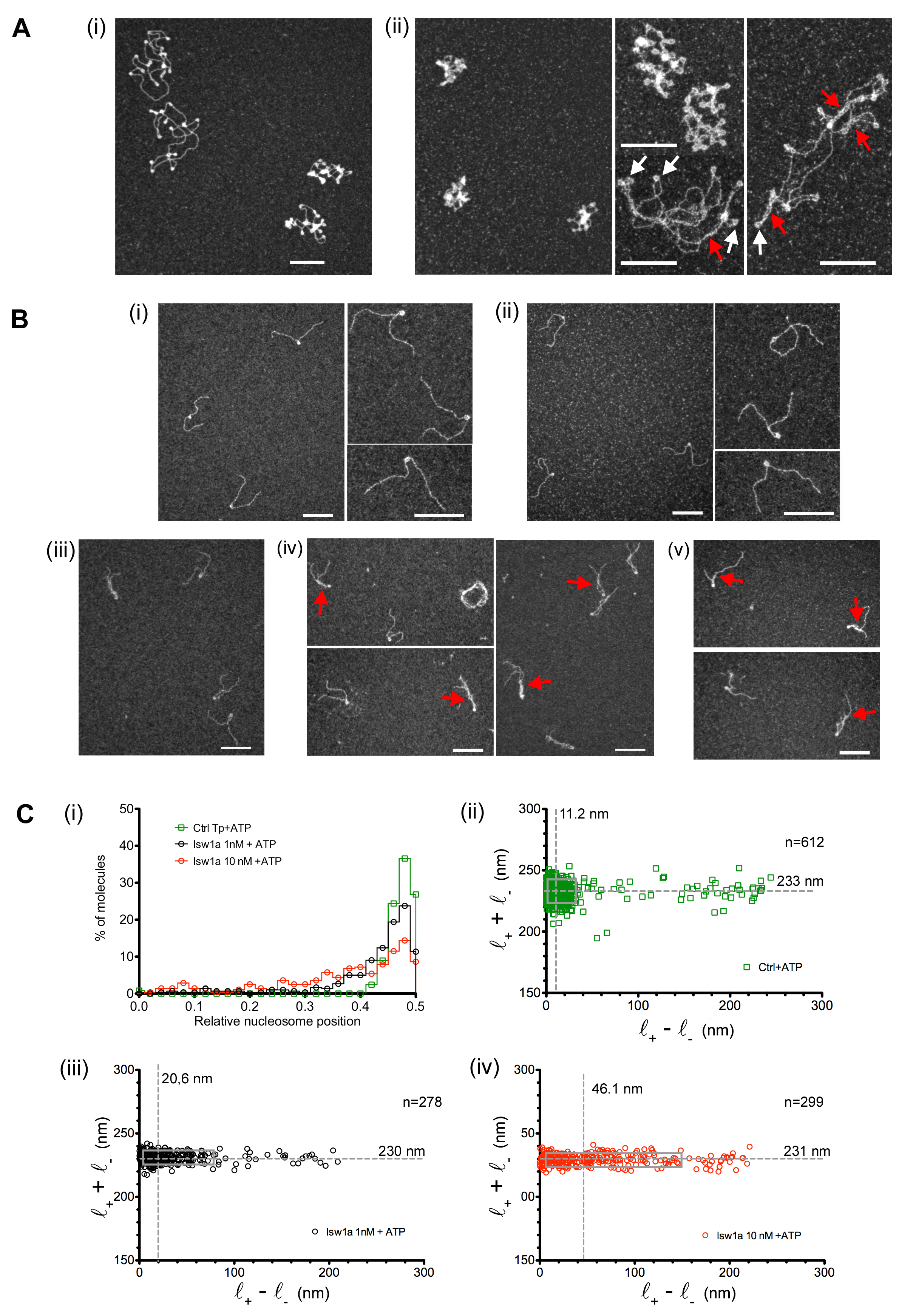

Supplement: Figure S6 — TEM analysis of Isw1a binding on chromatinized plasmid DNA. (A) (i) Representative image of phiX174-RFI plasmid (750 nM in bp) (New England Biolabs) chromatinized with nucleosomes from calf thymus core particles in binding buffer with 100 µM ATP and 2 mM MgOAc for 20 minutes at 30°C without (left) and with 10 nM Isw1a (right panels). Bar represents 100 nm. (ii) Cooperative binding of Isw1a on nucleosomal arrays tend to compact chromatinized DNA and to bridge arms (red arrows) at the entrance and exit of the nucleosomes. White arrows show nucleosomes that can be clearly identified at apexes. (B) Representative images of mononucleosomes on 845 bps DNA containing a histone positioning 601 sequence: in absence (i, ii) or presence (iii–v) of Isw1a (1 nM (iii) or 10 nM (iv, v)), and in absence (i, iii, iv) or presence (ii, v) of 75 µM ATP. Binding or Isw1a is observed after 10 min incubation at 30°C (iv, right panel) and over 30 min incubation (iv, left panel). Scale bar represents 100 nm. (C) Effect of Isw1a concentration on remodeling efficiency. Nucleosome position on 845-bps 601 mononucleosomal substrates upon remodeling by Isw1a at 10 nM, 1 nM or in control protein buffer in 20 mM Tris-HCl pH 8.0, 30 mM KCl, 2 mM Mg(OAc)2 and ATP (75 µM) for 20 minutes at 30°C. (i) Relative nucleosome position probabilities calculated as the ratio of (l −)/(l ++l −). (ii, iii, iv) Dot plots of total length of extranucleosomal DNA (l ++l −) as a function of the difference of length between the longest and shortest DNA arm (l +−l −): (ii) control buffer+ATP (open green squares); (iii) Isw1a 1 nM+ATP (open black dots); (iv) Isw1a 10 nM+ATP (open red dots). Dotted grey lines represent median for each measure. Grey rectangles represent 10–90 percentiles. n is the number of molecules analyzed as a result of 2 independent experiments. (TIF) [file pone.0031845.s006.tif]

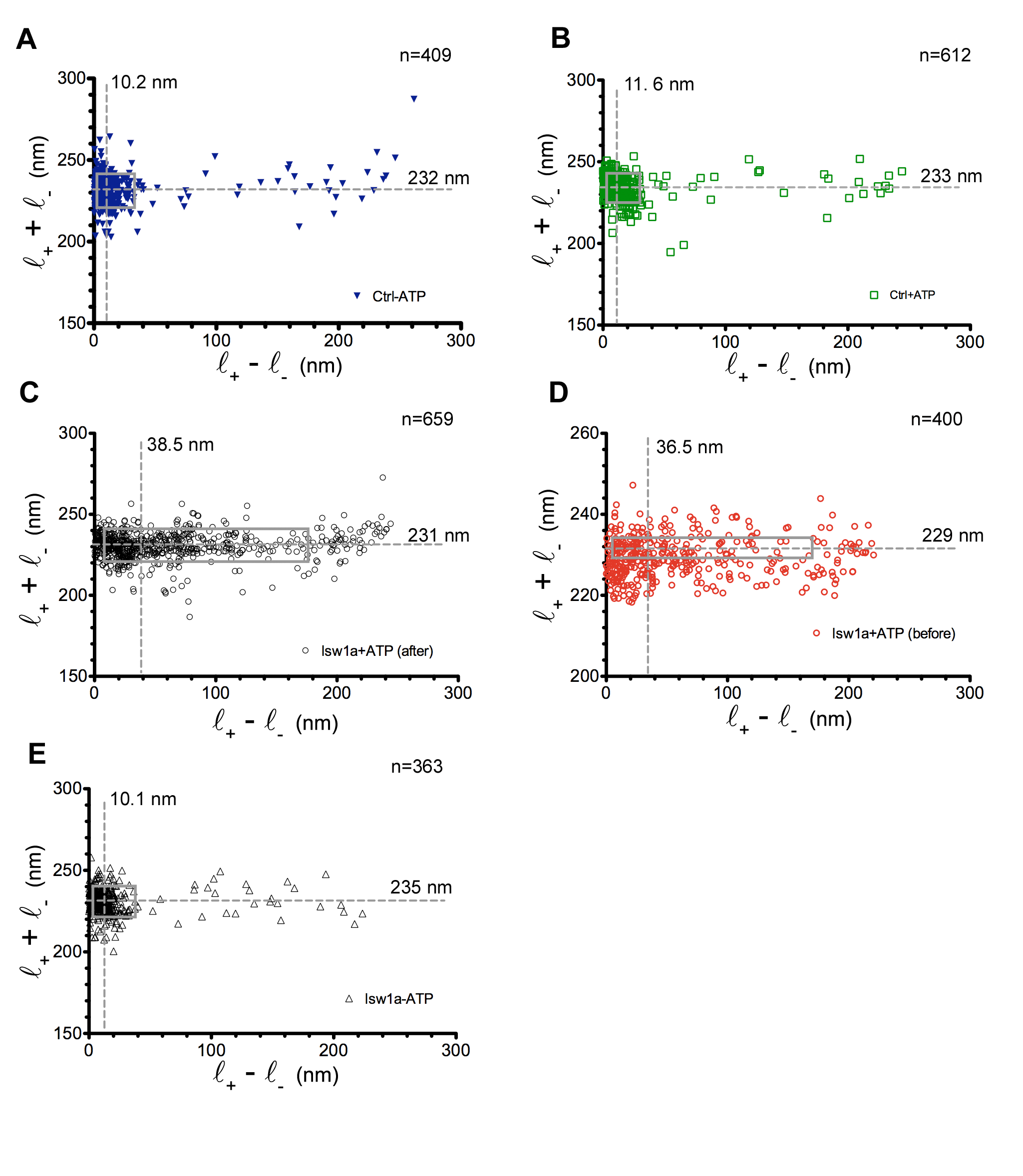

Supplement: Figure S7 — Plots of total length (ℓ++ℓ−) as a function of the difference between the longest and the shortest DNA arms (ℓ+−ℓ−). (A) Control without ATP (blue triangles); (B) control with ATP (open green squares); (C) ATP added after Isw1a binding for 10 min (see Figure S6B (iv, right)) (open black circles); (D) ATP added before Isw1a (open red dots); (E) Isw1a without ATP (open black triangles); Dotted grey lines represent median for each measure. Grey rectangles represent 10–90 percentiles. n is the number of molecules analyzed as a result of 2 (graph C (ATP after Isw1a)), 3 (graphs E and A) or 5 (graphs D (ATP before Isw1a) and B) independent experiments. (TIF) [file pone.0031845.s007.tif]

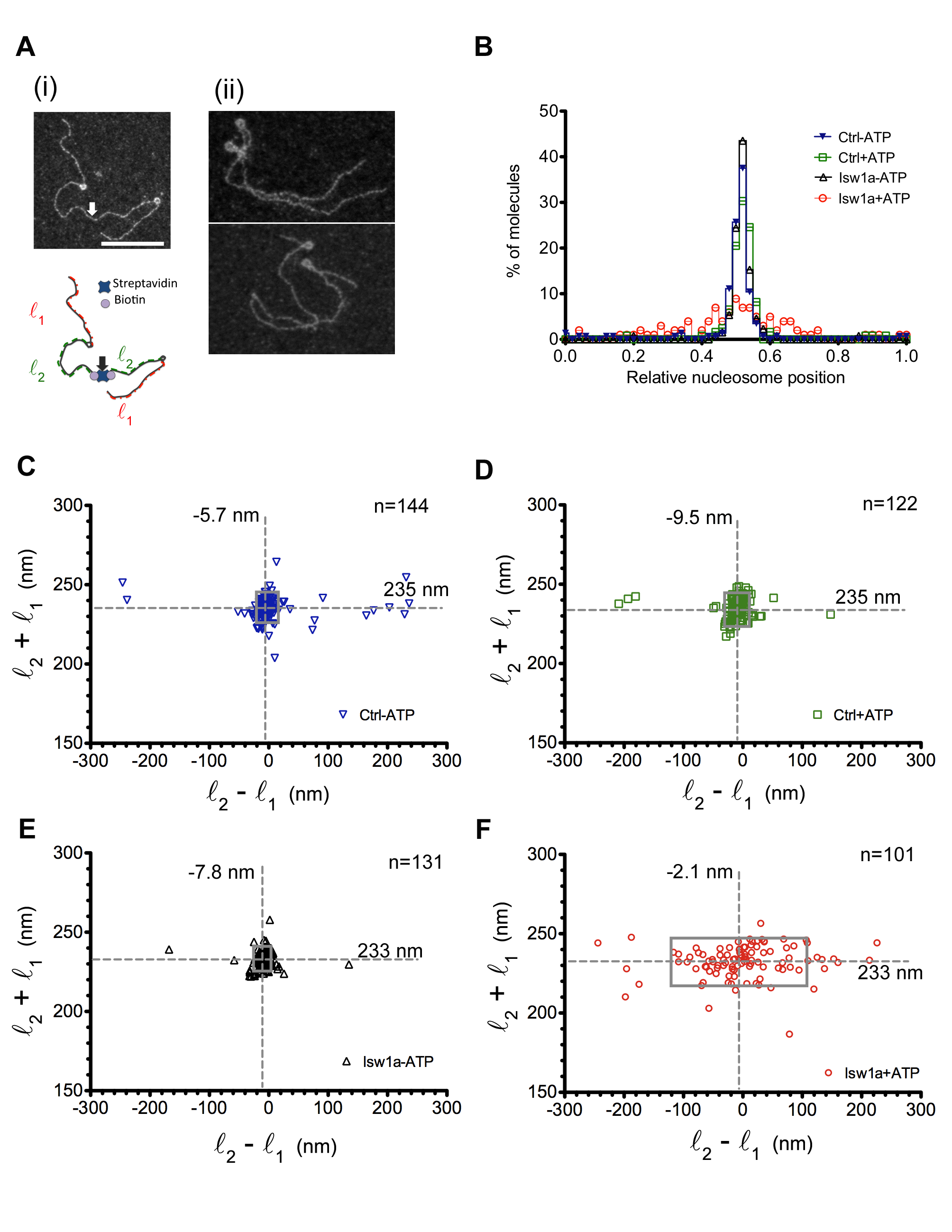

Supplement: Figure S8 — Binding to a dimer of biotinylated-845 bps mononucleosomal DNAs and remodeling activity of Isw1a. (A) Image of the two biotinylated-845 bps mononucleosomal DNAs (containing a histone positioning 601 sequence) dimerized by streptavidin (for molecule orientation) and schematic drawing representing the lengths measured in the analysis: in this oriented experiment ℓ1 represent the length from the nucleosome to the free end, ℓ2 the length from the biotinylated end to the nucleosome (i). Representative images of mononucleosomes on 845 bps DNA with 10 nM of Isw1a with of 75 µM of ATP (ii). Scale bars represent 100 nm. (B–F) Quantifications of nucleosome positions on the dimer of 845 bps mononucleosomal DNA. (B) Relative nucleosome position probabilities calculated as the ratio of (ℓ1)/( ℓ1+ℓ2). (C–F) dot plots of total length (ℓ2+ℓ1) as a function of the difference between the longest and the shortest DNA arms (ℓ2−ℓ1). (C) (open blue triangles) control without ATP; (D) (open green squares) control with ATP; (E) (open black triangles) Isw1a without ATP; (F) (open red dots) ATP added before Isw1a. Dotted grey lines represent median for each measure. Grey rectangles represent 10–90 percentiles. n is the number of molecules analyzed. (TIF) [file pone.0031845.s008.tif]
