# Supplementary material for: ATP-Independent Cooperative Binding of Yeast Isw1a to Bare and Nucleosomal DNA
Source: PLoS One. 2012 Feb 16;7(2):e31845. doi: 10.1371/journal.pone.0031845 (PMC3281020; doi:10.1371/journal.pone.0031845)
Supplement: Data S1 — Estimation of equilibrium constant kD. (DOCX) [file pone.0031845.s009.docx]

In conditions (F ~ 1 pN) where single binding /unbinding events can rarely be observed we could measure the average time in the on and of state (<τ_on_>, <τ_off_>). From the ratio of these average times, we can deduce [[1](#_ENREF_1)] the free energy of nucleation at zero force ∆G_0_:

*$\Delta G_{0}=k_{B}T\ln\left(\frac{\tau_{on}}{\tau_{off}}\right)+F\delta_{i}$* (2)

From the data of Figure S3B we ﬁnd that ΔG_0_ ~ 26pN nm = 6.3 k_B_T (where k_B_ is Boltzmann constant and T the temperature). Since in this experiment the concentration C_Isw1a_ ~ 0.5 nM, we deduce that the affinity for DNA of a single protein complex (at zero tension) is: k_D_ = C_Isw1a_ exp(−ΔG_0_/k_B_T ) ~ 1pM.

**SUPPLEMENTARY REFERENCES**

1. Lia G, Bensimon D, Croquette V, Allemand JF, Dunlap D, et al. (2003) Supercoiling and denaturation in Gal repressor/heat unstable nucleoid protein (HU)-mediated DNA looping. Proc Natl Acad Sci U S A 100: 11373-11377.
